# Natural and Biological Products Therapeutic Potential in Restoring Gut Microbiome: Mechanistic Insights and Clinical Implications

**DOI:** 10.1007/s12602-025-10796-9

**Published:** 2025-11-25

**Authors:** Shanmukha K, Vasavi Pasupuleti, Krishna Yadav, Shubhamkumar M Baviskar, Madhulika Pradhan, Lalitkumar K. Vora, Dharmendra Kumar Khatri

**Affiliations:** 1 Molecular & Cellular Neuroscience Lab, Department of Pharmacology and Toxicology, National Institute of Pharmaceutical Education and Research (NIPER), Hyderabad, Telangana India; 2 Rungta College of Pharmaceutical Sciences and Research, Kohka Road, Kurud, Bhilai, Chhattisgarh 490024 India; 3 School of Pharmacy, Rungta International Skills University, Bhilai, Chhattisgarh 490024 India; 4 Department of Pharmacology, NIMS Institute of Pharmacy, NIMS University Rajasthan, Jaipur, 303121 India; 5 School of Pharmacy, Queen’s University Belfast, 97 Lisburn Road, Belfast, BT9 7BL UK

**Keywords:** Gut microbiome, Probiotics, Prebiotics, Synbiotics, Herbal and dietary fibres, Safety recommendations

## Abstract

The gut microbiome is a complex ecosystem of trillions of microbes residing in the human gastrointestinal tract. This microbial community plays a pivotal role in human health by swaying various physiological processes like metabolism, immunomodulation, antimicrobial protection, and psychological health. Natural and biological products comprising herbal extracts, dietary fibers, probiotics, prebiotics, and synbiotics have long been documented for their potential to modulate gut microbiota composition and function. Understanding the interactions between natural products and gut microbiota can provide valuable insights into their health-promoting effects and therapeutic potential. Literature was systematically sourced, analyzed, and compiled through searches across databases such as Science Direct, Google Scholar, and PubMed. The search terms include “gut microbiome,” “Dysbiosis,” “probiotics,” “prebiotics,” “synbiotics,” “biological products,” “gut health,” “natural products,” “dietary fibers,” “resistant starches,” “clinical study reports,” “safety recommendations,” etc., and several combinations of these words. This article provides a deep insight into the overview of natural and biological products aimed at promoting a healthy gut microbiome. It underscores the functional roles of the gut microbiota, including nutrient metabolism, antimicrobial defense, and gastrointestinal tract integrity. Furthermore, it delves into the detailed mechanisms of action and clinical evidence supporting the use of probiotics, prebiotics, synbiotics, herbal extracts, and dietary fibers in modulating gut microbiota composition and function. The biological activity, detailed mechanism of action, safety, and their considerable potential as modulators of gut microbiota composition and function have been extensively studied. Further research is necessary to clarify the underlying mechanisms of action to investigate their clinical implications.

## Introduction

In circa 400 B.C., the venerable Hippocrates, a sage in the annals of medical history, pronounced profound wisdom when he declared, “death sits in the bowels” and discerned that “bad digestion is the root of all evil.” These prescient utterances underscored the pivotal role of the human intestinal milieu in fostering well-being. Within the domain of human physiology, our attention is directed towards the complex domain of the gut microbiota, commonly referred to as gut flora. This assemblage of microorganisms has established their presence within our gastrointestinal (GI) tract. This thriving ecosystem, comprising a staggering assembly of approximately 100 trillion microorganisms, predominantly of bacterial origin, has firmly entrenched itself within the human colon [228]. However, it is important to acknowledge the existence of a smaller population of eukaryotic species, such as fungi, protozoa, and viruses, that live within the favorable environment of the human intestinal ecosystem. The epicenter of microbial activity within the human GI domain resides in the large intestine, where anaerobic bacteria reign supreme [117]. This enclave represents one of nature’s most densely populated and diverse bacterial ecosystems. However, it is worth noting that the microbial composition within the human colon exhibits considerable variation among individuals, as elucidated by the exhaustive endeavors of the Human Microbiome Project [70, 192]. The microbial journey begins in utero, with a lifelong odyssey characterized by dynamic shifts in population and diversity. Notable milestones along this odyssey include pivotal transitions during breastfeeding and the introduction of solid foods. Microbial communities fluctuate not only in quantity but also in taxonomic makeup and functional attributes as they traverse the GI tract [1]. In the domain of herbivores, the ruminants—comprising creatures such as cattle, sheep, goats, alpacas, and deer—confront a distinct dietary challenge. Lacking the enzymatic artillery necessary for dismantling recalcitrant plant materials like cellulose and hemicellulose, ruminants have fostered a symbiotic alliance with an array of bacteria, protozoans, and fungi [4]. This alliance takes residence in their four-chambered stomach, known as the rumen, reticulum, omasum, and abomasum. Here, these microorganisms embark on the arduous task of breaking down the fibrous plant materials, rendering them digestible for their host. Subsequently, partially or fully digested fare journeys through the small and large intestines, where the host’s digestive machinery comes into play. Intriguingly, the anatomical and functional characteristics of the ruminant’s small intestine closely mirror those of humans and other mammals, albeit proportionally elongated—often reaching lengths up to 12–30 times the animal’s body length [4]. Delving deeper into the bays of scientific inquiry, we must recognize the burgeoning recognition of gut microbiota as a veritable reservoir of therapeutic potential. A plethora of research endeavors has illuminated the profound interplay between gut microbiota and human health, spanning various life stages, from infancy to adulthood [182]. Moreover, investigations have unveiled the pivotal role of gut microbiota in diverse maladies, including inflammatory bowel diseases, cardiometabolic disorders, cancer, and neuropsychiatric afflictions (Gomaa, 2020a). Mechanistically, the microbiota orchestrates a synchronization of signals that trigger cytokine production, thereby shaping the development and maturation of the host’s immune apparatus (Y. [140, 141, 143]). The symbiotic reciprocity of this relationship is encapsulated succinctly by the axiom, “Feed your microbiota and get fed by it” [239].

Dysbiosis, a state marked by the perturbation of gut microbiota and its functions, can arise from a multitude of etiological factors, including dietary indiscretions, sedentary lifestyles, stressors, aging, pharmaceutical agents, and xenobiotics [97]. A wealth of evidence buttresses the linkages between dysbiosis and a spectrum of maladies, notably those afflicting the gastrointestinal domain, such as ulcerative colitis, inflammatory bowel disease, colorectal cancer, and Crohn’s disease. This dysbiotic interplay extends its pernicious influence on extra-intestinal terrain, fomenting disorders like obesity, diabetes, cardiovascular maladies, and associated micro- and macrovascular complications. Remarkably, the pathogenesis of metabolic disorders may be instigated by the malfunctioning innate immune system, a scenario catalyzed by microbial modulations [97]. Turning our discerning observation toward nutrition, we discern a multifaceted role for diet, extending beyond the provision of sustenance to encompass a realm of bioactive molecules known as “nutraceuticals.” These compounds, found within foods of varying chemical compositions and functionalities, transcend mere nutritional utility. They confer health benefits when consumed regularly, endowing food products with the epithet “functional foods” [245]. This category encompasses non-nutrient entities such as probiotics and phytochemicals, along with macro- and micro-nutrients, exemplified by fatty acids and vitamins. Dietary habits wield significant sway over the composition of gut microbiota in humans, herbivores, and mice alike [91]. Long-term dietary proclivities exert substantial influence over this dynamic microbial milieu, with consequential repercussions on the absorption of nutraceuticals, micronutrients, and vitamins, thereby reshaping the delicate equilibrium of the intestinal ecosystem [91].

Among the pantheon of nutraceuticals, phytochemicals emerge as non-nutrient compounds imbued with potent biological activity. Synthesized through the primary and secondary metabolism of plants, phytochemicals pervade fruits, vegetables, seeds, nuts, whole grains, legumes, dark chocolate, and tea [212]. These bioactive molecules, although relatively few in terms of identified and isolated species, number in the tens of thousands across the plant kingdom. Notably, phytochemicals exert their salutary effects by selectively modulating the intestinal microflora, a phenomenon referred to as “probiotic” action. This comprises the nurturing of bacterial populations in the gut, including endosymbionts like yeast, bifidobacterium, lactic acid bacteria, and bacilli, integral to the metabolic intricacies of the human and animal gastrointestinal tracts [212]. Taxonomically, phytochemicals may be classified into several categories, including polyphenols, alkaloids, terpenoids (comprising carotenoid terpenoids and non-carotenoid terpenoids), organosulfur compounds, and nitrogen-containing compounds [147]. This classification mirrors the biosynthetic origins of these compounds. Chief among these are polyphenols, a group endowed with manifold virtues, including anti-inflammatory properties, the ability to impede cancer cell proliferation, the reduction of carcinogenic agent formation, modulation of gene expression, hormone signaling, immune potentiation, DNA protection, oxidative cell defense, and the activation of insulin receptors [147]. The bioavailability of phytochemicals, however, is curtailed within the human body, owing to their intricate chemical structures and their status as xenobiotics. Paradoxically, this poor absorption culminates in their prolonged residence within the intestinal domain, where they exercise their beneficial influence by sculpting the microbial landscape [212, 279]. In the past two decades, a burgeoning body of evidence has illuminated the intricate interplay between dietary phytochemicals and GI microbial populations, unveiling the underlying mechanisms that underscore their salutary effects on both extra-intestinal and GI disorders [212]. Yet, there persists a lacuna, beckoning for a comprehensive review to elucidate the contemporary insights into the impact of phytochemicals on human gut microbiota and their mechanistic involvement in metabolic diseases. This endeavor shall not only pave the way for their judicious integration into human diets but shall also shed brief light on their effect on animal gut microbiota modulation [212, 279].

In modern times, two seismic shifts have reshaped the landscape of intestinal microbiology and immunology. Firstly, it has been incontrovertibly demonstrated that gut microbes serve as master architects, orchestrating a profound influence over the host’s internal environment. Secondly, the composition and metabolic outputs of these intestinal microorganisms exert an outsized impact on the host’s immune responses [269]. Thus, the manipulation of intestinal microorganisms has emerged as a novel avenue for nurturing health and fortifying immunity, captivating the attention of scholars and scientists alike. The human immune system stands as a bastion against foreign incursions, constituting two principal branches—innate and adaptive immunity [269]. Innate immunity, the guardian at the gate, operates as a swift and unspecific initial line of defense, demanding no prior development for its effector functions. In contrast, adaptive immunity, a more refined and versatile arm of defense, refines its recognition repertoire through growth and maturation, enabling the discernment of self from non-self-antigens. This multifaceted system hinges on the orchestrated interplay between T and B lymphocytes and antigen-presenting cells, facilitating immune memory formation, immune homeostasis maintenance, and the elicitation of pathogen-specific immune responses [269]. Notably, research has underscored the intricate interplay between gut microbiota and both innate and adaptive immunity. For instance, studies have unveiled how flagellin, an immune-stimulating molecule, can trigger an adaptive immune response, guided by the compass of innate immunity. The microbial ecosystem even exerts control over flagella production, furthering the maintenance of mucosal integrity and homeostasis [115]. Furthermore, investigations have illuminated the relationship between adaptive immune responses and the composition of intestinal microbiota, a nexus with far-reaching implications in fending off formidable diseases. Indeed, the undeniable role of gut microbiota in sculpting the entire immune system has ushered in a new era of understanding [115].

In the biological orchestration, probiotics emerge as dietary influencers, capable of molding the human gut microbiota and, by extension, sculpting the composition and structure of the intestinal microbial consortium. It bears emphasis that intestinal flora serves multifarious roles within the human body. It not only fortifies the integrity of the mucosal barrier but also acts as a font of nourishment and a bulwark against pathogenic intruders [89]. The intricate relationship between immunity, sleep, and skin character has piqued recent scientific interest. Moreover, the central nervous system, beholden to the sway of the immune system, finds itself inexorably influenced by the actions of intestinal flora [89]. In this intricate web of interactions, immunity emerges as an omnipresent sentinel, directly shaping the contours of human existence. The judicious deployment of probiotics, a potent tool in regulating gut bacteria, stands as an effective strategy for enhancing immunity.

**Fig. 1:**
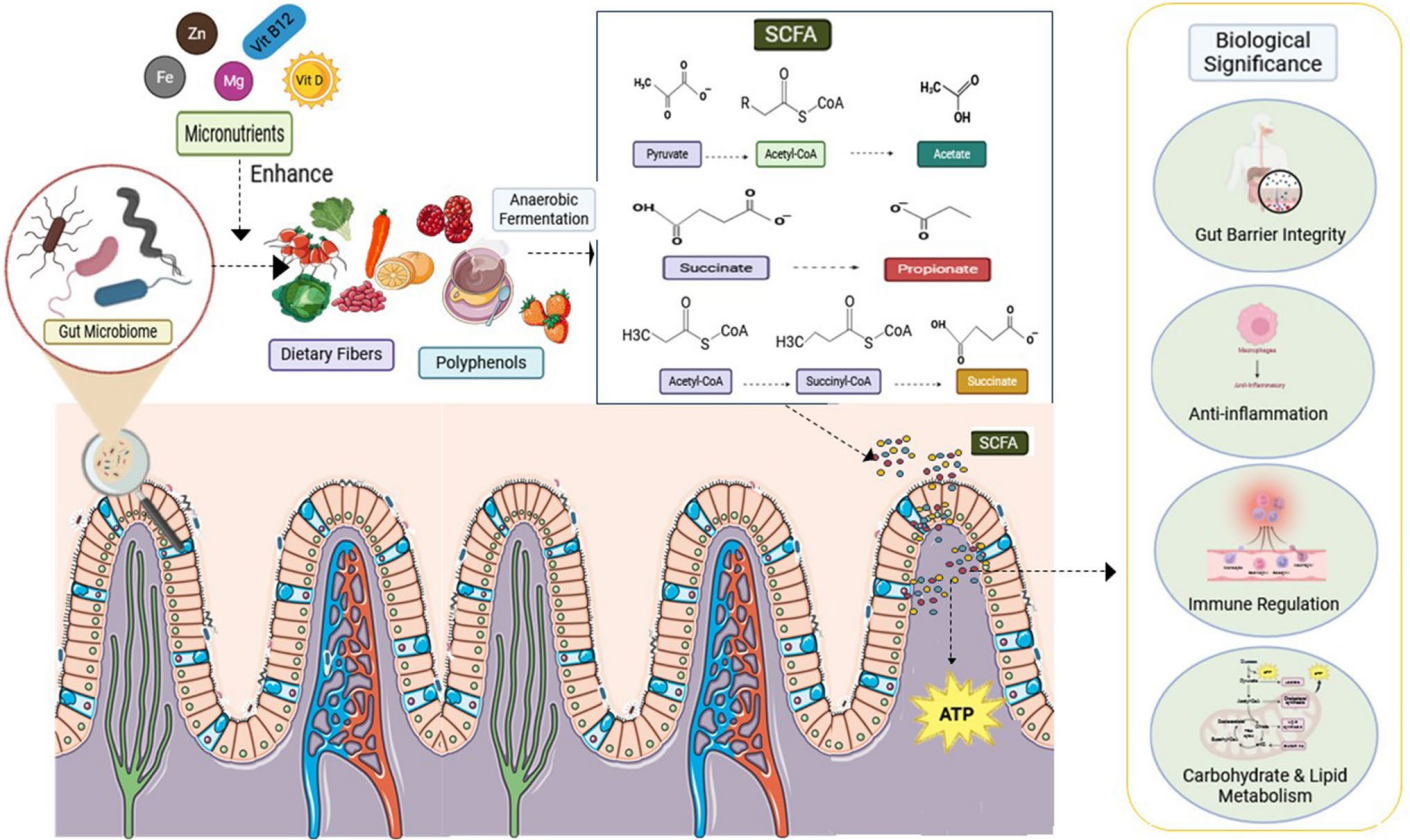
Illustration of the gut microbiota system and its role in the conversion of natural products into micronutrients

This review delves deeply into the domain of natural and biological agents tailored for the cultivation of a harmonious gut microbiome while underscoring the pivotal role this microbiome assumes in the panorama of holistic health as depicted in Fig. 1. The central aim resides in the judicious evaluation of these agents’ capacity to cultivate an exuberant gut microbiota, thereby fostering an overarching state of well-being. The exposition initiates its voyage by forging a robust foundational comprehension of the gut microbiome. This encompasses a rigorous dissection of its constitution, multifaceted functions, and the intricate milieu of determinants that govern its flourishing. Subsequent sections meticulously scrutinize an array of natural and biological products. Probiotics, prebiotics, synbiotics, herbal interventions, dietary fiber, and resistant starches are subjected to intricate scrutiny. Their respective mechanisms of action, coupled with the clinical substantiation that underpins their utility, are unearthed and delineated with precision. Simultaneously, the discourse exercises vigilance in addressing safety contingencies, encompassing potential adverse effects, contraindications, and potential interactions with conventional medications and adjunctive supplements. This pragmatic approach culminates in the formulation of judicious directives for practical application, thereby minimizing unwarranted risks. The compendium culminates with a resounding synthesis, encapsulating the verdict on the efficacy of these natural and biological agents while projecting their ramifications onto the broader landscape of forthcoming research and clinical praxis. In sum, this exegesis serves as an indispensable compendium for the discerning researcher, healthcare luminary, and individuals avidly seeking to orchestrate a state of optimal gut health through the strategic integration of natural and biological resources.

## Understanding the Gut Microbiome

### Definition and Composition of the Gut Microbiome

In the intricate domain of the gut microbiota, we encounter an expansive network teeming with approximately 100 trillion microorganisms. This assembly comprises an assortment of bacterial, viral, fungal, archaeal, and protozoal inhabitants, all establishing their presence within the confines of the gastrointestinal tract [245]. Notably, these microorganisms collectively manifest as a distinctive organ system, actively engaging in complicated dialogues with luminal antigens within the intestinal tract and the delicate lining of the intestines. Their influence, however, extends far beyond these boundaries, forging connections, both direct and indirect, with an array of vital organ systems, including the immune system, the endocrine system, and the intricate nervous system [27].

What makes the gut microbiota even more remarkable is its adaptability. The composition of this microbial consortium is not cast in stone; rather, it undergoes a perpetual transformation along the entire length of the gastrointestinal tract. This metamorphosis is orchestrated by a multitude of factors, encompassing genetic predispositions, dietary preferences, environmental influences, and the therapeutic interventions of medications. Astonishingly, this adaptability is not confined to long-term changes but also encompasses short-term fluctuations in response to daily rhythms, including circadian and diurnal cues [2].

**Fig. 2:**
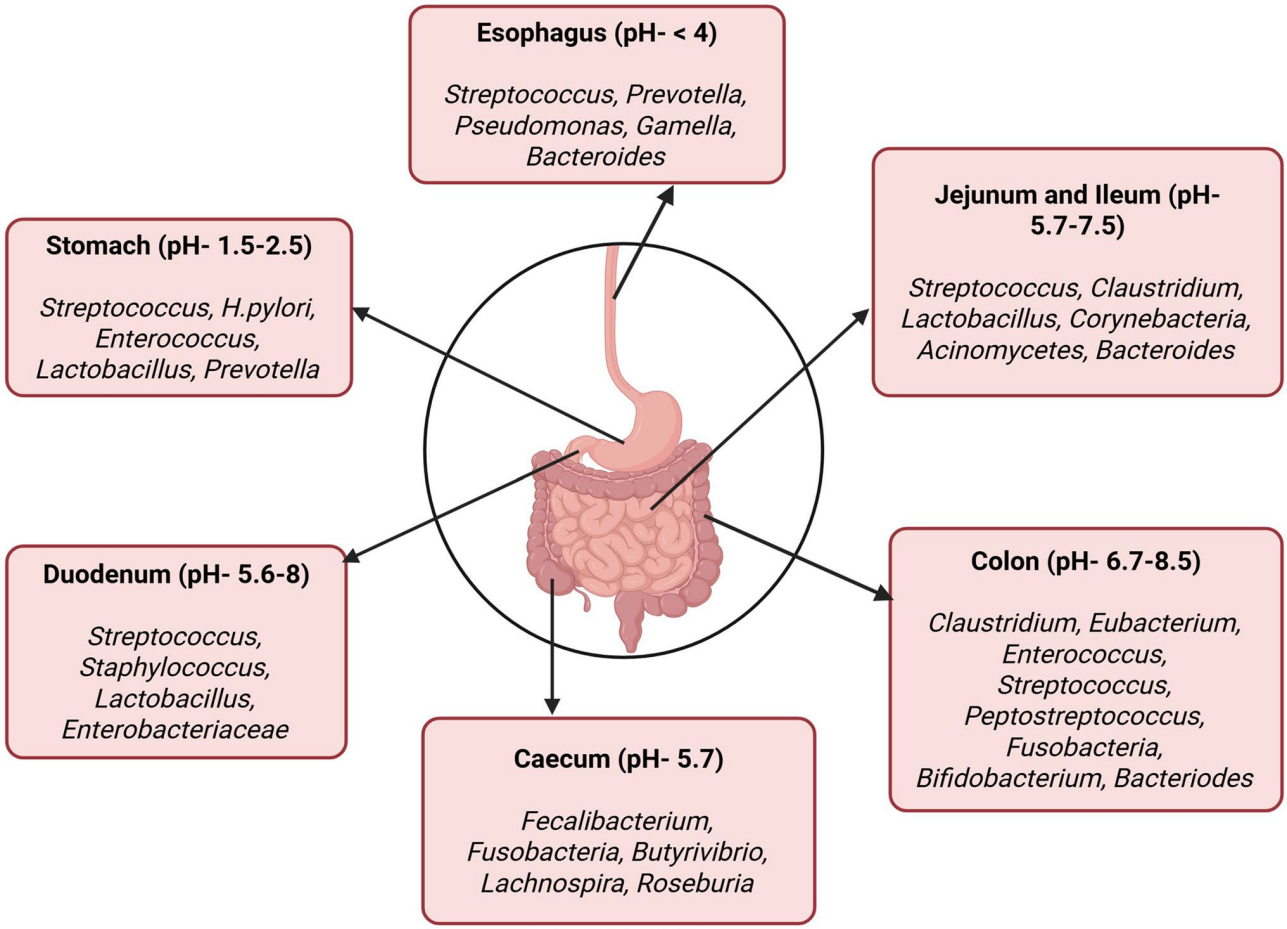
Dispersal of the normal human gut flora. Pictographic imaging starting from the esophagus traveling down to the colon spotlighting, different bacterial strains along this path

In general, healthy gut microbiota is primarily composed of the phyla Firmicutes and Bacteroidetes, followed by *Actinobacteria* and *Verrucomicrobia*. While this broad pattern remains consistent, the distribution of gut bacteria varies both over time and along the digestive tract, with significant differences in diversity and bacterial counts as moving from the esophagus down to the colon and distal gut as depicted in Fig. 2 [292].

In the distal esophagus, duodenum, and jejunum, Streptococcus reigns as the dominant genus. However, once we enter the stomach, Helicobacter takes center stage and shapes the entire microbial landscape. When *Helicobacter pylori* (*H. pylori*) coexists in the stomach as a friendly neighbor, we find a diverse community featuring other prominent genera like *Streptococcus*, *Prevotella*, and *Veillonella.* This diversity diminishes if *H. pylori* turns pathogenic. Now, we focus on the large intestine, which houses over 70% of all the microbes in our body. Here, two major phyla, Firmicutes and Bacteroidetes, dominate the scene. Traditionally, the ratio of Firmicutes to Bacteroidetes was thought to be linked to disease susceptibility, but recent research has unveiled significant variability even among healthy individuals, casting doubts on its relevance (Gomaa, 2020b).

Beyond Firmicutes and Bacteroidetes, the human colon accommodates primary pathogens like *Campylobacter jejuni*, *Salmonella enterica*, *Vibrio cholera*, and *Escherichia coli*, along with *Bacteroides fragilis*. However, these pathogens remain in low abundance, making up 0.1% or less of the entire gut microbiome. The presence of Proteobacteria is scarce, and its absence, coupled with the high presence of signature genera like *Bacteroides*, *Prevotella*, and *Ruminococcus*, indicates a healthy gut microbiota. Moving from the gut’s lumen to the mucosal surface, we see differences in microbial populations. While luminal microbial genera such as *Bacteroides*, *Bifidobacterium*, *Streptococcus*, *Enterobacteriaceae*, *Lactobacillus*, *Enterococcus*, and *Clostridium* are prominent (detectable in stool), the mucosa and mucus-associated genera mainly include *Clostridium*, *Lactobacillus*, *Enterococcus*, and *Akkermansia* (found in the small intestine) [284].

In essence, the gut is a diverse microbial world where different genera and species play distinct roles, influenced by factors like location in the digestive tract, health status, and individual variation.

### Functional Aspects

These distinctive qualities of the gut microbiome have propelled scientific inquiry from a focus on the sheer abundance and diversity of microbial residents to a keen interest in their functional roles. In the following section, we embark on a concise journey to unveil the primary functions performed by these stalwart members of the normal gut microbiota. This section provides a brief overview of the major functions of the normal gut microbiota.

#### Nutrient Metabolism

Carbohydrates, mainly found in foods like grains (such as corn, wheat, and oats), stand as a crucial component in pig feed, constituting 60 to 70% of the entire feed. These carbohydrates take different paths in the body depending on their characteristics, like how easily they ferment and dissolve in water. Water-soluble carbohydrates get broken down by digestive enzymes, absorbed in the small intestine, and used throughout the body. On the other hand, insoluble carbohydrates (like non-starch polysaccharides, fibers, and resistant starch) cannot be digested in the small intestine. Instead, they journey through the large intestine and get fermented by gut microbes [165]**.**

From this fermentation, short-chain fatty acids are produced and can be absorbed by various cells in the body. Butyrate, a type of short-chain fatty acid, provides about 10 to 15% of the body’s energy needs and helps repair damaged gut lining. Acetate and propionate, other short-chain fatty acids, play roles in liver energy metabolism. Butyrate also helps prevent the buildup of harmful metabolic by-products like d-lactate. Bacteria from the Bacteroides genus are champions in carbohydrate metabolism, producing enzymes like glycoside hydrolases, glycosyl transferases, and polysaccharide lyases. Among them, *Bacteroides thetaiotaomicron* is a standout with over 260 hydrolases in its genome, far more than the human genome. These microbes, along with species like *Oxalobacter formigenes*, *Lactobacillus*, and *Bifidobacterium*, help counter the formation of kidney stones by dealing with oxalate produced during carbohydrate fermentation [198]. Researchers have shown that different carbohydrates can influence gut microbes, affecting aspects like food intake, digestion, nutrient absorption, metabolism, and the host’s physiology [164]**.**

For instance, a recent study found that high levels of raw potato starch intake in the context of a typical American diet may modify vulnerability to gastrointestinal bacterial infections [224]. Similar effects were observed by partially hydrolyzed guar gum and it was reported that guar gum effectively altered gut microbiota of weanling piglets and promoted body weight accretion and health [100]**.**

Pectin, another dietary component, is believed to support the growth and activity of beneficial bacteria like *Prevotella*, *Lactobacillus*, and *Faecalibacterium*, promoting gut health. The gut microbiota also has a system for managing proteins. It uses its enzymes, like proteinases and peptidases, alongside the body’s enzymes. These enzymes help break down proteins into smaller molecules. The microbes on the surface of bacteria transport amino acids from the gut into the bacteria. Inside these bacteria, various genes collaborate to convert these amino acids into small signaling molecules and protective proteins called bacteriocins. Our gut microbiota also aids in producing vitamins like vitamin K and some B vitamins. Specific bacteria like Bacteroides species generate a beneficial fatty acid called conjugated linoleic acid, known for its health benefits [176].

Furthermore, the gut microbiota, including bacteria like *Bacteroides intestinalis*, *Bacteroides fragilis*, and *E. coli*, can transform primary bile acids into different forms, called secondary bile acids. These changes primarily occur in the colon and have significant health implications [24].

Recently, scientists have discovered that the gut microbiota plays a crucial role in breaking down various natural compounds called polyphenols. These polyphenols are present in many foods, including fruits, plants, and beverages like tea and wine. They encompass different types such as flavonoids, tannins, and others. Inside the gut, these polyphenols convert into other substances that may have various health benefits [185].

As we delve deeper into the world of the gut microbiota, we uncover a fascinating realm of tiny organisms that work in harmony with the body. This collaboration involves complex processes contributing to overall health and well-being. Continued exploration of this intriguing world aims to benefit humanity.

#### Xenobiotic and Drug Metabolism

In the realm of our digestive system, ingested xenobiotics encounter the thriving communities of microorganisms residing in the small intestine and colon. These microorganisms possess a remarkable ability to alter these xenobiotics in ways that are either distinct from or complementary to the host’s own bodily processes. Considering human metabolism, it predominantly involves three key mechanisms: oxidation, hydrolysis, and the bonding of chemicals with small molecules like glucuronide or glutathione. In sharp contrast, the metabolic capabilities of gut bacteria are extraordinarily diverse. These microorganisms primarily modify substances through reduction reactions, the addition of acetyl and methyl groups, and the creation of radicals [53]**.**

The profound implications of microbial influence become apparent when considering its impact on the fate of xenobiotics within the body and, consequently, overall health. The gut microbiome possesses the capacity to either disrupt or accelerate the way the body processes these foreign substances, affecting how they are distributed, metabolized, and eliminated. Despite the current understanding of how the gut microbiome can significantly alter the pharmacokinetics of xenobiotics, this knowledge has not yet been fully embraced in the realm of drug development [123].

In the ensuing years, a burgeoning body of evidence has shed light on the pivotal role played by the gut microbiota in drug metabolism. The microbiome’s influence extends to compounds such as cardiac glycosides, exemplified by digoxin. Notably, recent findings have illuminated how these compounds prompt the upregulation of a cytochrome-containing operon within the common organism *Eggerthella lenta*, hailing from the *Actinobacteria phyla*. This, in turn, culminates in the inactivation of digoxin, exemplifying the microbiome’s direct impact on drug efficacy [136].

The metabolism of drugs in the stomach by bacteria is a problem for therapeutic efficacy and safety, and it can be difficult to predict in people. The cardiac glycoside digoxin, a therapy for heart failure and arrhythmia, is an intriguing example of a drug being digested by a single bacterium. Digoxin works by directly attaching to and inhibiting Na +/K + ATPases via its unsaturated lactone ring, lowering Ca^2+^ content in cardiac myocytes [42]**.**

Another captivating illustration of microbiome-induced drug metabolism revolves around the anticancer agent irinotecan. Here, the microbial enzyme β-glucuronidase catalyzes the deconjugation of irinotecan, contributing to the drug’s undesirable side effects, including diarrhea, inflammation, and anorexia [33]. These intricacies illuminate the multifaceted role of gut microbiota in drug metabolism and underscore the potential significance of this relationship in shaping the landscape of therapeutic interventions for various diseases.

#### Antimicrobial Protection

Maintaining the delicate balance of a healthy gut is a complex task for the immune system within the gut mucosa. It must coexist with beneficial microbes while preventing harmful ones from taking over. To achieve this, a multifaceted defense system comes into play.

A two-tiered mucus layer protects the large intestine. This dynamic structure protects luminal bacteria from intestinal lining interaction. Mucin glycoproteins from intestinal goblet cells make up mucus. This reaches up to 150 μm from the colonic epithelium. The dense inner layer lacks organisms, while the active outer layer feeds microorganisms with glycans. Additionally, goblet cells produce trefoil-factor and resistin-like molecule-β, which stabilize mucin polymers and maintain barrier integrity [110].

In contrast, the small intestine employs a different strategy. Here, the mucus layer is discontinuous and less effective. As a result, antimicrobial proteins (AMPs) play a more significant role. The gut microbiota, through its structural components and metabolites, stimulates the host’s Paneth cells to synthesize AMPs. These AMPs are crucial for reinforcing mucosal barrier function. Notably, Paneth cells, located in the base of small intestinal crypts, concentrate AMPs in this region. While a diverse and healthy microbiota appears necessary for AMP production, specific species like *Bacteroides thetaiotaomicron* and *Lactobacillus innocua* play key roles. *Lactobacillus* species, which create lactic acid, interact in another intriguing way. This acid disrupts the bacterial cell wall’s outer membrane, boosting host lysozyme’s antibacterial effectiveness. Besides these complex AMP expression pathways, bacterial metabolic metabolites including short-chain fatty acids and lithocholic acid can trigger cathelicidin expression via histone deacetylation. AMPs mostly destroy commensal and pathogenic bacteria surface structures [106].

The gut microbiota has also evolved an effective strategy to control the overgrowth of harmful strains: it induces local immunoglobulins. Especially, Gram-negative organisms like *Bacteroides* activate intestinal dendritic cells, to stimulate plasma cells in the intestinal mucosa to produce immunoglobulins. These immunoglobulins coat the gut microbiota and make it resistant to degradation by bacterial proteases.

#### Immunomodulation

The microbiota, especially those in the gut, play an important role in shaping the host’s immune system by regulating both local and systemic immune responses. The inner lining of the gut, known as the gut mucosa, contains numerous types of intestinal epithelial cells, including goblet and Paneth cells, and is just under the lamina propria.

Goblet cells help by creating a mucus layer that acts as a protective barrier. Through steric hindrance, it prevents microbial attachment to intestinal epithelial cells and acts as a decoy for microbial adhesion. Surprisingly, the presence of bacteria is required for the development of this mucus layer, as evidenced by the absence of it in germ-free (GF) mice [99].

On another note, Paneth cells step into action as guardians against unwanted microbial invaders. They do so by releasing antimicrobial peptides, preserving the pristine condition of the inner mucus layer. Just beneath the surface of the intestinal epithelial cells, an energetic community of immune cells thrives within the lamina propria. This vibrant congregation includes antigen-presenting cells, such as dendritic cells, as well as T cells and B cells.

Microbes, true to their nature as initiators, initiate localized immune responses by engaging with immune cells equipped with pattern recognition receptors (PRRs), like Toll-like receptors. This engagement triggers a cascading effect for dendritic cells, propelling them on a migratory expedition from the gastrointestinal tract to the mesenteric lymph nodes (mLNs). Within the mLNs, dendritic cells unveil antigens derived from microbes, guiding the transformation of naive T cells into specialized effector T cells. Among these, two distinguished categories come to the fore: regulatory T cells (Tregs) and T helper 17 (Th17) cells [246].

A subset of these effector T cells embarks on a journey back to the gastrointestinal tract, where they wield their influence over local immune responses. Simultaneously, another contingent enters the broader circulation, leaving an imprint on systemic immunity. Tregs, the epitome of balance, play a pivotal role in orchestrating an immune system shift, steering it away from inflammation and towards an anti-inflammatory state. They execute this vital task by releasing anti-inflammatory cytokines like IL-10 and TGF-β or through their interactions with dendritic cells.

In stark contrast, Th17 cells chart a course that tilts the immune system towards a pro-inflammatory stance. They accomplish this feat by secreting immunostimulatory cytokines such as IL-17 or by mobilizing and enlisting neutrophils. Interestingly, the lamina propria of GF mice stands devoid of these pro-inflammatory Th17 cells. Nevertheless, their resurgence is catalyzed by a specific cohort of bacteria known as segmented filamentous bacteria. This intricate interplay underscores the pivotal role of microbes in the activation of Th17 cells [69]**.**

As we delve further into the intricate tapestry of host-microbe interactions, we unveil a narrative where microbial influence profoundly shapes the course of immune responses, both locally and systemically. This symbiotic dance between microbes and the immune system remains an enchanting and vital chapter in the story of human health, a narrative continually enriched by ongoing research and discovery.

#### Maintenance of Integrity of the Gastrointestinal Tract

In the present scientific landscape, a substantial body of evidence firmly establishes the pivotal role that the gut microbiota plays in preserving the structure and functionality of the gastrointestinal tract. One remarkable illustration of this synergy is found in the endocannabinoid system, which orchestrates the gut microbiota’s involvement in upholding the functionality of the gut barrier. A noteworthy example comes from *Akkermansia muciniphila*, a Gram-negative bacterium capable of elevating endocannabinoid levels, thus wielding control over the functions of the gut barrier and mitigating metabolic endotoxemia [215].

Additionally, the gut microbiota actively contributes to the development of the structural aspects of the gut mucosa. This intricate process is orchestrated by the induction of the transcription factor angiogenin-3, renowned for its pivotal role in shaping the intestinal microvasculature. This concept finds validation in the observations made in GF mice, which exhibit a substantial reduction in the capillary network within the villi. Such reductions can lead to compromised digestion and absorption of nutrients. Furthermore, the evidence stemming from GF mice showcases a reduced intestinal surface area, thinner villi (attributed to impaired regeneration), prolonged cell cycle times, and disrupted peristalsis, collectively underscoring the profound impact of the gut microbiota on the structural dynamics of the gastrointestinal tract [73]**.**

Moreover, the gut microbiota exercises precise control over the patterns of mucosal glycosylation. These patterns serve as crucial anchoring points for microbes, operating at both the cell surface and subcellular levels. For instance, a signaling molecule produced by the microorganism *Bacteroides thetaiotaomicron* acts as a catalyst, triggering the expression of the carbohydrate structure known as fucose on cell surface glycoconjugates. This intricate interplay paints a vivid portrait of the multifaceted role played by the gut microbiota in preserving the structural and functional harmony of the gastrointestinal tract [268]**.**

### Factors Influencing Gut Microbiome Health

There are several intrinsic and extrinsic factors that can influence the composition of the gut bacteria and ultimately affect health.

#### Age

While conventionally held beliefs suggest that the colonization of the gut by microorganisms commences immediately after birth, intriguing emerging evidence hints at the possibility of microbial presence even during fetal development. Examination through 16S rRNA-based sequencing techniques has unveiled the initial composition of the infant’s meconium, a newborn’s earliest stool, to be enriched with genera like *Escherichia-Shigella*, *Enterococcus*, *Leuconostoc*, *Lactococcus*, and *Streptococcus* [162, 170].

Yet, it becomes evident that this inaugural microbial profile is substantially influenced by the method of delivery. In instances of vaginal births, the infant’s intestines initially receive colonization from microorganisms sourced from the maternal vagina, most notably represented by the genera *Lactobacillus* and *Prevotella*. Conversely, in cesarean deliveries, the infant’s gut tends to be primarily colonized by microbes from the maternal skin flora, typified by the prevalence of *Streptococcus*, *Corynebacterium*, and *Propionibacterium* [41].

The initial composition of the infant’s gut microbiota, following this primary inoculation, appears transient and characterized by a lack of diversity. However, over time, it stabilizes, diversifies, and eventually attains a composition bearing 40 to 60% similarity with that of an adult by the age of 3. Nevertheless, studies have unveiled that young children and adolescents may exhibit significant variations in the proportions of Bacteroides and Bifidobacterium compared to adults [170].

#### Diet

The earliest influence on the composition of the gut microbiota, following the manner of a baby’s birth, pertains to the initial diet provided to the infant—either breast milk or formula. Research has unveiled notable disparities in the gut microbial makeup between infants nourished through breastfeeding and those relying on formula feeds. This divergence becomes increasingly significant as contemporary mothers are increasingly opting for non-breastfeeding practices. Diet maintains its significance throughout adulthood as the chief determinant shaping the composition, diversity, and richness of the gut microbiota. Broadly, diets rich in fruits, vegetables, and fibers are linked to greater richness and diversity of gut microbiota. Individuals adhering to such dietary patterns display increased levels of insoluble carbohydrate-metabolizing organisms within the Firmicutes phylum [18].

Moreover, even short-term dietary modifications wield substantial influence on the gut microbiota. For example, a mere 4-day shift to an animal-based diet has been shown to diminish the abundance of Firmicutes while elevating the presence of bile-tolerant organisms from the phylum Bacteroidetes and Proteobacteria [132]**.**

Significant variations in the gut microbiome across geographical regions and seasons have also been documented. Notably, these variations align closely with distinct dietary patterns. For instance, rural African children exhibit a higher prevalence of Prevotella, indicating an agrarian diet, while European children display increased proportions of Bacteroides, indicative of a Western diet rich in animal protein and lacking in fiber [18]**.**

Dietary polyphenols, besides their systemic antimicrobial and metabolic functions, can inhibit specific gut bacteria. For instance, quercetin, a polyphenolic compound, is degraded by certain bacteria, including *Bacteroides*, *Enterococcus*, and *Eubacterium* species. Conversely, hesperetin, another polyphenol, exhibits limited degradation by colonic microbiota and exerts inhibitory effects on vancomycin-intermediate *Staphylococcus aureus* and *H. pylori* [258].

Seaweeds, brimming with bioactive compounds, offer diverse biological benefits such as antibacterial, antioxidant, anti-inflammatory, anti-coagulant, anti-viral, and apoptotic activities. Rich in soluble fibers and sulfated polysaccharides, they influence the gut microbiota significantly. Studies in both humans and rats have demonstrated notable shifts in gut microbiota upon seaweed supplementation. This suggests the potential utilization of seaweeds as effective prebiotics, fostering a favorable gut microbial environment [281]**.**

#### Genetics

Genetics plays a significant role in shaping the composition of the gut microbiota, impacting host metabolism and, consequently, overall health. It has been observed that family members tend to share more similarities in their microbiota communities compared to unrelated individuals. Additionally, monozygotic twins, who share identical genetic makeup, exhibit more similar gut microbiota than dizygotic twins [21]**.**

Despite these insights, there are currently no comprehensive genome-wide studies that have pinpointed specific genes and pathways responsible for determining the precise composition of the gut microbiome. However, it is worth noting that certain genes within the immune system have been linked to conditions like inflammatory bowel disease, shedding light on the interplay between genetics and the gut microbiota in health and disease [193].

#### Infections

The gut microbiota has a dual influence on infections, both viral and bacterial. For instance, when investigating an infection caused by *Citrobacter rodentium* in mice, researchers discovered that this infection led to changes in the gut microbiota, particularly a decrease in the presence of Lactobacillus and other bacterial groups. In humans, a study involving patients with *Clostridium difficile* infection and asymptomatic carriers revealed that both groups had reduced microbial richness and diversity compared to healthy individuals, indicating a significant impact of this bacterial infection. *Clostridium difficile* infection often arises due to severe disruptions in the gut microbiota [46, 264].

Interestingly, a clinical approach involves transplanting the gut microbiome from healthy donors to infected patients, which has been shown to increase microbial richness and diversity, offering a promising therapeutic avenue. Moreover, research using a mouse model of hepatitis B virus infection demonstrated that establishing a balanced gut microbiota is crucial for effectively clearing the hepatitis B virus. Alterations in the composition of the host’s gut microbiota influence both the development and resolution of bacterial and viral infections [290]**.**

#### Antibiotics

Although research on antibiotics has predominantly focused on their abilities to kill or inhibit the growth of harmful microorganisms, recent years have witnessed a growing body of work exploring their impact on the complex ecosystem of the gut microbiota. This holistic perspective has revealed that the use of antibiotics carries both short-term and long-term consequences for the normal gut microbial community.

Strong evidence suggests that multi-drug-resistant bacterial genes predate antibiotics and were caused by environmental exposure to inhibitory chemicals. An unbalanced commensal microbiome may also produce resistance genes. This disruption breaks the host’s intestinal environment’s positive interaction with a healthy gut flora. A crucial defense mechanism employed by the healthy gut microbiota against pathogens is competitive exclusion. Antibiotics were shown to disrupt this protective mechanism several decades ago, leading to infections by pathogens like Salmonella. One possible mechanism for this disruption is the breakdown of the complex web of interactions among different species within the microbiota. This breakdown can increase the levels of host-derived sialic acid, providing a PPRfavorable growth environment for pathogens such as *Clostridium difficile* and *Salmonella typhimurium* [180].

Exposure to antibiotics has been shown to exhibit both immediate and long-lasting changes in the naturally occurring microorganisms in our bodies. The effects of antibiotics on the gut microbiota include reduced taxonomic diversity and persistent changes in a significant portion of individuals. Even short-term use, such as a 7-day course of broad-spectrum antibiotics like Clindamycin, can have lasting impacts, with the diversity of Bacteroides species failing to fully recover for up to 2 years [195].

A brief eradication therapy targeting *H. pylori* with clarithromycin-containing triple therapy reduced *Actinobacteria* diversity and increased the ermB resistance gene 1000-fold. This effect lasted over 4 years in some individuals, although others recovered. Ciprofloxacin, which targets Gram-positive bacteria, has a short-lived effect on *Ruminococcus* species [74, 106].

However, a significant concern arises considering the implications of employing broad-spectrum antibiotics. Beyond their impact on altering the diversity of microorganisms residing in the gut, these antibiotics potentially fuel the dissemination of antibiotic-resistant strains through a process known as horizontal gene transfer. This complex mechanism allows bacteria to exchange mutant genetic information across different species. What adds a layer of intrigue to this scenario is that the human gut microbiota is remarkably prone to horizontal gene transfer, exhibiting a likelihood 25 times higher than other environments. Consequently, it underscores the utmost importance of exercising caution and prudence in the utilization of broad-spectrum antibiotics, lest we inadvertently contribute to the proliferation of antibiotic resistance within this delicate microbial ecosystem [180].

## Natural and Biological Products for Promoting Gut Microbiome Health

### Overview of Natural and Biological Products

Natural and biological products are gaining attention in therapeutic approaches to promote gut microbiome health with fewer side effects. Growing evidence suggests the effects of these products on the gut microbiome. Natural products like herbs, whole grains, vegetables, fruits, functional foods (like yogurt, kefir, sauerkraut, kimchi, miso, tempeh, kombucha, natto, pickles, traditional buttermilk, and some fermented vegetables) and biological products like *Lactobacillus*, *Bifidobacterium*, *Saccharomyces*, *Streptococcus*, *Bacillus*, *E. coli*, and *Enterococcus* help in maintaining digestive health, boost the immune system, improve mental health, and lower the risk of infections.

### Probiotics

Probiotics is a Latin/Greek term meaning “for Life.” In 1907, a Russian scientist, Elie Metchnikoff, published a scientific rationale for Lactic acid bacteria (LAB) having health benefits (“Metchnikoff: The prolongation of life—Google Scholar,” n.d.). Probiotics are live beneficial microorganisms such as bacteria or yeast that when consumed in adequate amounts provide a health benefit to the host. In 2001, the International Scientific Association of Probiotics and Prebiotics (ISAPP) consensus on the definition with The Food and Agriculture Organization of United Nations and World Health Organization (FAO/WHO); Panel Experts reaffirmed the major principles of the term probiotic [196, 206]. These live microbes can colonize the gut and improve digestive health by replenishing a balanced gut microbiome in GI health [11],Wang et al., 2021a).

#### Types of Probiotics

Numerous studies proved that *Lactobacillus*, *Bifidobacterium*, *Saccharomyces*, and other genera including *Bacillus*, *Escherichia*, *Streptococcus*, *Enterococcus*, and *Propionibacterium* could be used as probiotics (Table 1). Probiotics encompass this diverse variety of strains that help to regulate the immune system and gut health. Below is a list of some common bacterial strains accepted as probiotics [47, 61]. The lactic acid bacteria group consists of vast strains of Gram-positive, facultative anaerobic rod-shaped bacteria (including *Lactobacillus*, *Lactococcus*, and *Enterococcus*), which secrete lactic acid as byproducts in carbohydrate metabolism [92, 163, 223, 287]. *Bifidobacterium*, possessing anti-microbial activity, is “generally recognized as safe” gram-positive, non-motile obligate anaerobes which are endosymbiotic inhabitants in the GIT and vagina of mammals. *Bifidobacteria* strains like *B. breve* and *B. pseudocatenulatum* help in pediatric and preterm infant diseases like diarrhea, infant colics, celiac disease, obesity, allergic, and neurodegenerative diseases [209, 218, 267]. The bacterium Lactococcus aids in lactose digestion and bolsters intestinal health [142, 277], and improves immunomodulatory actions [205]. *E coli*, gram-negative bacteria, when non-pathogenic, acts as a probiotic assisting metabolic functions (*Escherichia coli* Nissle 1917) [213, 286]. *Bacillus* strains produce digestive resilience and affect immune balance. The strains like *B. substilis BS50*, *B. coagulans LBSC,* and B. coagulans Unique IS2 proved the reducing symptoms of constipation and IBS [67, 150, 183]. *Saccharomyces* (yeast) has the potential to improve gut health and immune fortification. *Saccharomyces boulardii* and *Saccharomyces cerevisiae* are the strains that showed effectiveness in GI diseases like infectious diarrhea, constipation, colitis, and IBS [55, 65, 166].

**Table 1:** Types of probiotics and prebiotics

Probiotic genus	Common sources	Strains of probiotics	Ref
*Lactobacillus*	Yogurt, kefir, Pickles, sauerkraut, miso, and fermented foods	*L. acidophilus*, *L. rhamnosus*, *L. reuteri*, *L. gasseri*, *L. casei*, *L. plantarum*, *L. johnsonii*, *L. gallinarum*, *L. salivarius*, *L fermentum*, *L. brevis*,* L. amylovorous*	[47, 163, 226]
*Bifidobacterium*	Dairy products, sourdough bread, and other fermented foods	*B. infantis*, *B. longum*, *B. lactis*, *B. animalis*, *B. breve*, *B. adolascentis*, *B. bifidum*,* B. pseudolongum*	[47, 159]
Yeast	Yeast-based supplements	*Saccharomyces boulardii*, *S. carlsbergensis*, *Kluyveromyces marxianu*, *S. cerevisiae*,* S. lactis*	[47, 179]
Other microorganisms	Dairy products and other fermented foods	*Bacillus subtilis*, *B. coagulans*, *B. cereus*, *B. licheniformis*, *Enterococcus faecalis*, *E. faecium*, *Leuconostoc mesenteroids*, *L. citreum*, *L. lactis*, *Pediococcus acidilactici*, *P. pentosaceus*, *Streptococcus salivarius subsp. Thermophilus*, *S. infantarius*,* Propionibacterium freudenreichii*	[5, 47]
**Prebiotics**			
**Prebiotics**	**Glycosidic bond**	**Degree of polymerization**	**Ref**
Inulin	β-(2 → 1)	2–60	[25]
Fructo-oligosaccharide (FOS)	β-(2 → 1)	2–9	[25]
Galacto-oligosaccharides (GOS)	β-(1 → 3), β-(1 → 4) and β-(1 → 6)	2–8	[25]
Xylooligosaccharide (XOS)	β-(1 → 4)	2–7	[25]
Lactulose	β-(1 → 4)	2	[25]
Human-milk oligosaccharide (HMO)	β-(1 → 3), β-(1 → 4) and β-(1 → 6)	2–8	[20]

#### Mechanism of Action in Promoting Healthy Gut Microbiome

Probiotic plays a vital role in fostering a healthy gut-microbiome [3], through diverse mechanisms of action, that include modulating the composition and diversity of the gut microbiome and can restore the microbial imbalances known as “dysbiosis” by elevating good microbes and preventing the proliferation of bad pathogens which is termed as Colonization and Competitive Exclusion. The beneficial mechanisms of probiotics include adhesion, intestinal epithelial barrier function, host immune system, and immune modulation [16, 145].

i. Colonization of Gut Microbial Communities and Competitive Exclusives with Pathogens

In the early life stages of birth, abnormal colonization in the gut microbiome affects growth and health, resulting in short-term and long-term adverse effects [37]. An assemblage of the gut microbiome develops in an infant’s gut, and there are several factors influencing the early intestinal microbial colonization patterns of preterm babies. These include newborn genetics, prenatal and maternal complications like postnatal medical conditions, feeding type, antibiotics usage, delivery mode, and inflammatory conditions [37, 186, 275]. Bacteria strains like *Enterococcus faecium*, *Staphylococcus epidermidis*, *Propionibacterium acnes*, and *Escherichia coli* are isolated from the umbilical cord, placenta, and meconium affect the colonization [109, 186]. Frequently used probiotic strains are *Bifidobacterium* spp. *B.breve* strain BBG-OO1 in NEC (Necrotising enterocolitis) in preterm infants [62], and *Bifidobacterium breve* M-16 V [267]. The prophylactic administration of the probiotic combination *Bifidobacterium bifidum* NCDO2203 and *Lactobacillus acidophilus* NCDO1748 improves neurodevelopment in neonates [14].

**Fig. 3:**
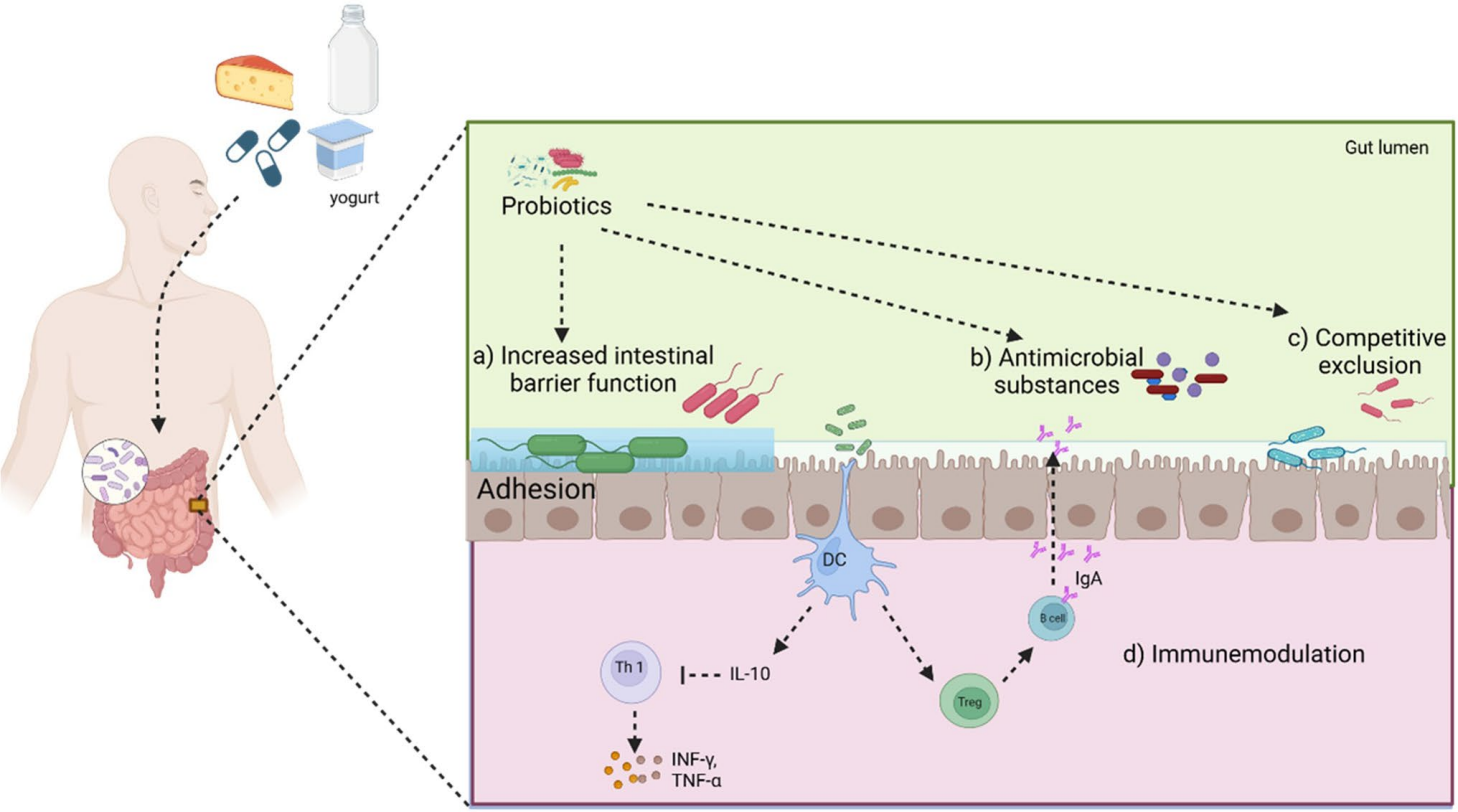
Schematic representation of mechanism of action of probiotics on healthy gut microbiome

*Lactobacillus reuteri* colonizes the host microbiome, by forming a biofilm that enhances colonization resistance [168] against uropathogens [163]. As portrayed in Fig. 3, probiotics exhibit a notable mechanism of competition by preventing the adhesion of pathogens to the intestinal surface. *Bacillus* probiotic eliminates dangerous *Staphylococcus aureus* by altering gene regulation and blocking the *S. aureus* signaling system [183]. Studies demonstrated some foodborne pathogens also affect competitive exclusion [167]. *Lactobacillus* strains prevent the adhesion of isolates of *Pseudomonas aeruginosa* in Cystic Fibrosis [13].

ii. Probiotic—Immunomodulatory Function

The benefits of probiotics in the defense mechanism of intestinal epithelium are by improved epithelial barrier function and secretion of defensins, mucins, trefoil factors, immunoglobulin A (IgA), cytokines, TLRs, gut-associated lymphoid, and signaling pathways [256]. Probiotics regulate intestinal flora and enhance host immunity (Wang et al., 2021b). Specific strains of *Lactobacillus* and *Bifidobacteria* are shown to enhance adaptive immunity, including T and B cells and memory development. It is also known to show modulatory effects by increasing natural killer cell cytotoxicity as well as macrophage phagocytosis, thereby influencing both adaptive and innate immunity [9]. As portrayed in Fig. 3, specific strains of probiotics show immunomodulatory effects, like strains of LAB that enhance interferon-gamma (IFN-γ) and promote the shift from Th2 to Th1, resulting in lowering the allergic reactions. *L. reuteri* and *L. casei* upregulate the activation of Treg cells and Interleukin-10, and exhibit anti-inflammatory effects. *L. rhamnosus* HN001in repressing NF-kB pathway and *S. boulardii* enhance transforming growth factor-beta (TGF-β) [9, 45].

iii. Probiotics—Intestinal Epithelial Barrier Function

Probiotics fortify the intestinal barrier function [288]. The probiotic surface consists of components such as pili, flagella, surface layer proteins (SLPs), capsular polysaccharides (CPs), lipopolysaccharide, lipoteichoic acid, and microbial-associated molecular patterns (MAMPs). They specifically bind to PRRs such as TLRs and NOD-like receptors. Probiotics are alternatives to antibiotics for the prevention/treatment of intestinal diseases. Articles documented that different probiotic species exert health benefits for the gut by playing a pivotal role in restoring intestinal microecology and intestinal mucosa [251]. LOCK strains of *Lactobacillus* spp. demonstrated that colonization in the gut with LOCK strains affects the maturation and formation of the epithelial gut barrier. They have a role in the formation of junctions between intestinal epithelial cells by activation of proteins (zonulin and occludin). Moreover, they increase the permeability of the intestinal barrier. Colonization of the strains strengthens the intestinal barrier and advances protection against pathogen/infectious agents by activating the secretion of IgA production as depicted in Fig. 3 [126].

iv. Antimicrobial Substances Production

Probiotic strains produce organic acids like lactic and acetic acids and low molecular weight chemicals (bacteriocin). Organic acids are regarded as primary anti-microbial substances due to their high inhibitory action on Gram-negative bacteria. They enter the cell in an undissociated form and accumulate inside the cell, causing pH imbalance resulting in antimicrobial activity. Bacteriocin and antimicrobial peptides are chemicals that exhibit a narrow activity by penetrating into cells or decreasing the cell wall synthesis of pathogens. These are mostly produced by LAB groups like *L. acidophilus* (lactation), *L. plantarum* (plantaricin), and *Lactococcus lactis* (nisin). Probiotic bacteria produce a variety of metabolic products that interfere with the growth of other toxic bacteria [251]. As portrayed in Fig. 3, metabolic products like short-chain fatty acids (SCFAs) modulate the gut microbiome and improve health benefits by damaging peptidoglycan [38]. Most studies demonstrated the beneficial effect of probiotics on intestinal microbiome balance by producing metabolites like SCFAs, B-vitamins, polyphenols, neurotransmitter precursors like serotonin and gamma-aminobutyric acid (GABA), and enzymes [25, 26, 157, 237]. SCFAs are important probiotic metabolites and are also produced by other gut microbes, through fermentation of dietary products in the colon. SCFAs include acetate, butyrate, and propionate. Their main function is to provide an energy source,colonic cells utilize SCFAs as an energy source and maintain the intestinal barrier by preventing the entry of toxic metabolites and pathogens by enhancing tight junctions between SCFA colonic epithelial cells [154, 291].

#### Evidence for Effectiveness and Clinical Trials

Probiotics have been extensively considered for their positive effects on GI disorders. Clinical studies have been done to prove evidence for the effectiveness in inflammatory bowel disease, ulcerative colitis, and irritable bowel syndrome (J. [118, 119, 128, 133, 166, 209]). Extensive clinical trials have been conducted on probiotics to investigate their effects on chronic conditions like asthma, allergic rhinitis, and neurodegenerative diseases [48],Farahmandi et al., 2022a; [204]. Several strains of probiotic bacteria like *Bifidobacterium breve*, *Lactobacillus*, *E. coli*, and *Saccharomyces* have been evaluated, out of which some of them were approved for their beneficial effects. The data of the probiotics, as mentioned above, along with the therapeutic indication and dosage specifications, are listed in Table 2.

**Table 2:** Summary of clinical evidence on probiotics, prebiotics, and synbiotics

Probiotic	Dose	Subjects	Clinical Study design	Health benefits	Ref
*Lactobacillus paracasei* HA-196 and *Bifidobacterium longum* R0175	Not specified	251 IBS patients	Randomized trial	Reduce the severity of GI symptoms in IBS patients	[133]
*Bifidobacterium pseudocatenulatum* CECT 7765	10^9–10^ c.f.u, daily for 13 weeks	48 obese children with insulin resistance	Interventional clinical study	Substantial reduction in body mass index	[209]
*Lactobacillus gasseri* BNR17	10^9^ c.f.u/day-low dose 10^10^ c.f.u/day-high dose for 12 weeks	90 subjects	Randomized, double-blind, placebo-controlled trial	Reduce visceral fat mass	(J. [118, 119])
*E. coli* Nissle 1917	200 mg once daily for about 12 months	327 patients	Randomized, double-blind, placebo-controlled trial	Reduce UC severity	[127]
*Bifidobacterium animalis* subsp. animalis IM386 and *Lactobacillus plantarum* MP2026	Not specified	44 patients	Randomized, placebo-controlled trial	A significant lowering effect on flatulence and diarrhea	[199]
*Lacticaseibacillus paracasei* nor *L. rhamnosus*	Not specified	1591 participants	Randomized, double-blind, placebo-controlled trial	Reduce symptoms of allergic rhinitis	(Farahmandi et al., 2022b)
*Lactobacillus* and *Bifidobacterium spp*	Not specified	2417 children	Randomized clinical trial	Reduces respiratory infections	[48]
*Bifidobacterium breve* CCFM1025	10^10^ c.f.u daily for 4 weeks	45 patients diagnosed with MDD (major depression disorder)	Randomized clinical trial	Minimizes depression and GI discomfort	[240]
*Lactobacillus Plantarum* 299v	Not specified	79 patients with MDD (major depression disorder)	Double blind, randomized placebo trial	Improved cognitive performance	[203]
*Saccharomyces cerevisiae* I-3856	8 × 10^9^ c.f.u daily for 8 week	456 subjects with IBS	Randomized, double-blind, placebo-controlled trial	Abdominal pain relief	[166]
*Lactobacillus casei* *L. acidophilus* *L. rhamnosus* *Bifidobacterium breve* *B. longum* *Streptococcus thermophilus*	3 × 10^9^ c.f.u/g each strain	40 asthmatic patients	Randomized, double-blind, placebo-controlled trial	Improved pulmonary function parameters and reduced inflammation	[204]
**Prebiotics**					
**Type of prebiotic**	**Study duration and dose**	**Subjects**	**Clinical study design**	**Microbiome modulation**	**Ref**
Inulin-type fructan (ITF)	6 g/day for 24 weeks	270 children (142 boys and 128 girls)	Randomized trial	*Bifidobacterium, Lactobacillus*	[144]
ITF	12 g/day for 4 weeks	Healthy adults with mild constipation	Randomized, double-blind, placebo-controlled trial	*Anaerostipes, Bifidobacterium*	[252]
Inulin	20 g/day for 42 days	12 non-diabetic adults with obesity	Randomized cross over trial	Promotes *Actinobacteria* and reduced *Clostridia*, *Clostridales*	[32]
FOS and GOS	16 g/day for 14 days	35 adults	Randomized controlled trial	*Bifidobacterium*	[139]
Short-chain GOS	1.5 to 15 g/day for 35 day treatment	85 lactose intolerance patients	Randomized, double-blind, placebo-controlled trial	Lactose fermenting *Bifidobacteria, Faecalibacterium* and *Lactobacillus*	[10]
GOS	Not exactly defined	20 adults	Randomized control	Increased *Bifidobacterium* and *Lactobacillus*,	[217]
FOS	probiotics + FOS (Dose not exactly defined)	26 children with ADS (Autism spectrum disorders)	Clinical trial	Increase in *Bifidobacteriales* and *B. longum*	(Y. [257, 260])
FOS	Three dose levels (2.5, 5, and 10 g/day)	80 healthy volunteers	Randomized, double-blind, placebo-controlled trial	Increased the relative abundance of OTUs belonging to *Bifidobacterium* and *Lactobacillus*	[232]
ITF	16 g of inulin-type fructans for 6 weeks	25 patients (15 men) aged 41–71 years	Randomized controlled trial	significant bifidogenic effect and induced increased concentrations of fecal SCFA	[19]
INF	15 g ITF/day for 2 weeks	26 healthy individuals	Single-group design trial	increased proportion of the *Bifidobacterium* genus, a decreased level of unclassified *Clostridiales*, and a decrease in *Oxalobacteraceae*	[94]
Bovine milk-derived oligosaccharides (MOS)	7.2 g MOS/L until the age of 6 months	114 infants	Double-blind, randomized, controlled trial	The strong *bifidogenic* effect reduces fecal pathogens and improves the intestinal immune response	[58]
Human milk oligosaccharides	5–23 g/L	infants	Randomized, double-blind, placebo-controlled trial	Increased *Bifidobacterium* and *Lactobacillus*	[238]
**Synbiotics**					
**Probiotic**	**Prebiotic**	**Subjects**	**Type of trial**	**Health benefits**	**Ref**
*Lactobacillus acidophilus*, *Bifidobacterium lactis, B. longum*, and *B.bifidume*	Galactooligosaccharides	20 human subjects	Clinical trial	Increased Bifidobacterium and Lactobacillus in the gut	
Probiotic capsule (*Bifidobacterium lactis, B. longum, B. bifidum, Lactobacillus acidophilus, L. rhamnosus*)	Short-chain FOS and encapsulated sodium butyrate	120 IBS patients	Randomized, double-blind, placebo-controlled trial	Decreased severity of symptoms of IBS	[68]
*Bifidobacterium breve*, *Lactobacillus casei*	Galactooligosaccharide	72 patients with sepsis condition	Randomized controlled trial	Reduce the chances of enteritis and VAP in sepsis patients	[221]
Kefir Grains	Fermented milk 2 mL/kg/daily for 90 days	AD patients	Clinical trial	Kefir appears to ameliorate the cognitive impairment	[241]
Synbiotic supplement		60 persons with excess body mass	Randomized trial	Intestinal bacterial diversity has increased, with the decrease in zonulin concentration in feces	[105]
Probiotics supplement	Fructooligosaccharide 500 mg for 10 weeks	56 adult hypothyroid patients	Randomized, double-blind, placebo-controlled trial	Observed an improvement in blood pressure	[194]
*Lacticaseibacillus paracasei* YIT 9029, *Bifidobacterium* YIT 12272	GOS (7.5 g)	88 obese patients with type 2 diabetes	Randomized controlled study	Increased levels of *Bifidobacterium* and *Lactobacillus*	[111]
*Bifidobacterium animalis* subspecies *lactis*	FOS 4 g twice per day for 10–14 months	104 patients	Randomized trial	Decrease in liver fibrosis indicators	[216]
*Bifidobacterium animalis* subsp. *lactis* 420™ (B420)	Letisse® Ultra™ polydextrose (LU)	134 obese adults	Randomized controlled trial	*Methanobrevibacter*, *Christensenellaceae*, and *Akkermansia* all increased with LU + B420, whereas Paraprevotella decreased	[93]
*Lactobacillus casei*, *L. plantarum*, *L. rhamnosus*, *Bifidobacterium lactis*	Zinc suspension 15 mg/day for 5 days	Children with acute infectious diarrhoea	Single-center, randomized and controlled clinical trial	Reduce diarrhoea symptoms	[278]
*Bifidobacterium longum* NT strain 1010 c.f.u/day	GOS 1 g/day	69 healthy adults with constipation tendency	Randomized, double-blind, placebo-controlled trial	Relief of constipation symptoms	[101]
Synbiotic supplement		135 overweight and obese women	Clinical trial	Improvements in body mass index and oedema volume	[244]
*Lactobacillus acidophilus*, *Bifidobacterirm bifidum*, *B. lactis* and *B. longum*	Inulin	87 participants	Randomized controlled trial	Reduce HDL levels and metabolic syndrome prevalence	[113]
Bovine milk fat globule membranes (MFGM),	Long-chain polyunsaturated fatty acids (LC-PUFA)	170 full-term healthy infants	Clinical study trial	Enhanced probiotic growth and delayed gut microbiota	(Cerdó et al., 2022b)
*Lacticaseibacillus paracasei* and *Bifidobacterium adolescentis and B. pseudocatenulatum*	GOS	88 obese patients with T2DM	Randomized clinical trial	Increased *Bifidobacterium* strains. Improve glucose metabolism and anti-diabetic effects	[111]
*Lactobacillus casei*	Inulin	32 subjects	Clinical trial	The oxidative stress indicators MDA, H2O2, and GSSG significantly decreased	[120]

### Prebiotics

In 1995, Glen R. Gibson and Marcel Roberfroid introduced the concept of “prebiotics” [72]. The term “prebiotic” is defined as “a non-digestible food constituent that has a positive impact by stimulating the growth of a limited group of bacteria on the host.” A small number of compounds of carbohydrates like fructans (fructo-oligosaccharides (FOS) and inulin), galactic-oligosaccharides (GOS), trans-oligosaccharides lactulose, and human-milk oligosaccharides (HMOs), also few fibers and endogenous enzymes, are documented as prebiotics. Prebiotics amplifies the group of beneficial bacteria genera like Bifidobacterium and Lactobacillus and also from other microbial taxa (e.g., *Roseburia*, *Faecalibacterium*, or *Eubacterium* spp.). The most common prebiotics employed are mannan-oligosaccharides, galactoglucomannans, inulin, lactose, and oligofructose. Short-chain carbohydrates contain three to ten sugar units (100). Prebiotics exhibit main characteristics like (a) resistance to gastric pH, hydrolysis by enzymes, and GI absorption; (b) fermentation by microflora lining of the intestine; (c) stimulation of growth/activity of the specific group of intestinal bacteria with health benefits [71]. Probiotics are non-digestible carbohydrates that can be of natural origin in plants and animals. For example, asparagus, garlic, sugar beet, onion, chicory, wheat, honey, fruits, vegetables, Jerusalem artichoke, human milk, seeds of lentils, legumes, beans, peas chickpeas, mallow, and mustard are rich in oligosaccharides [60].

#### Types of Prebiotics

##### i. Fructans

Fructans are found in various plants, including Jerusalem artichokes, globe, chicory, agave, bananas, wheat, onions, and garlic. Humans lack digestive enzymes to break down β-(2 → 1) linkage of fructans, whereas Lactobacillus and Bifidobacterium species of the gut microbiome produce 2,1-β-d-fructan hydrolase enzyme, aiding them to ferment fructans for nutrients. FOS, in particular, was the first fiber studied in the intestinal microbiome. Previous studies using FOS known as Neosugar, showed modulation of gut microbiota [88, 266].

##### ii. Inulin-Type Fructans

These are polysaccharides consisting of fructose molecules with a DP (degree of polymerization) ranging from 2 to 60. In other words, inulin is a single molecule linked with fructose units. As a prebiotic, it resists digestion in the upper GIT and reaches the colon. It serves as the substrate for the gut microbiome [98].

##### iii. Fructooligosaccharides

FOs are short-chain oligosaccharides composed of fructose units. FOs are found in natural sources including certain plant-based foods, onions, garlic, bananas, leeks, and asparagus. They are rapidly fermented in the colon, due to less short-chain length causing rapid production of SCFAs. Both inulin and FOs as prebiotic supplements contribute health benefits to the gut, immune function, balance of the gut microbiome, and GI tolerance (Sabater-Molina et al., 2009).

##### iv. Galactooligosaccharide

GOs are a type of prebiotic oligosaccharides known as oligo galactose, oligolactose, oligo galactosyl lactose, or trans-galacto-oligosaccharides (TOs). These are non-digestible carbohydrates, resistant to colon enzymes that are linked by glycosidic bonds of short chains of galactose units called TOs. In addition to *Bifidobacteria* and *Lactobacillus*, GOs also stimulate *Bacteriodetes*, *Enterobacteria*, and *Fermicutes* [66, 270]. This prebiotic fiber naturally occurs in various foods, legumes, other plant sources, and human breast milk and cow’s milk.

##### v. Xylooligosaccharides (XO)

XOs are a type of short-chain oligosaccharide, which consists of five carbon xylose units. These are stable to acidity and temperature. These are present in natural sources like bamboo shoots, vegetables, fruits, milk, and honey. Recent studies have demonstrated that XO supplementation alters the gut microbiota mostly the *Bifidobacterium* genus. XOs are selectively utilized by this genus, therefore acting as prebiotics. The XO structures like arabinoxylo-oligosaccharides (AXOs) and feruloylated arabinoxylo-oligosaccharides (FOXs) act with promising beneficial effects on specific gut microorganisms and may have potential benefits on metabolic health [6, 276].

##### vi. Human Milk-Oligosaccharides

HMOs are the most abundant complex group of carbohydrates present in human breast milk. These are the third important component after lactose and fats in human breast milk [20]. They play a pivotal role in infant health and development [255]. They are undigested carbohydrates, reach the infant’s intestine, and act as prebiotics by nourishing the gut-beneficial-bacteria, particularly *Bifidobacteria* spp., which are essential for infants’ digestive health and immune function. HMOs protect infants from sepsis and necrotizing enterocolitis (NEC), showing anti-inflammatory and antimicrobial properties [174],act as a barrier by preventing the attachment of pathogenic bacteria and viruses to intestine linings; and reduce the risk of GI infections in infants. HMOs also play a vital role in reducing allergies and autoimmune diseases by influencing the immune system [293].

#### Mechanism of Action for Healthy Gut Microbiome

**Fig. 4:**
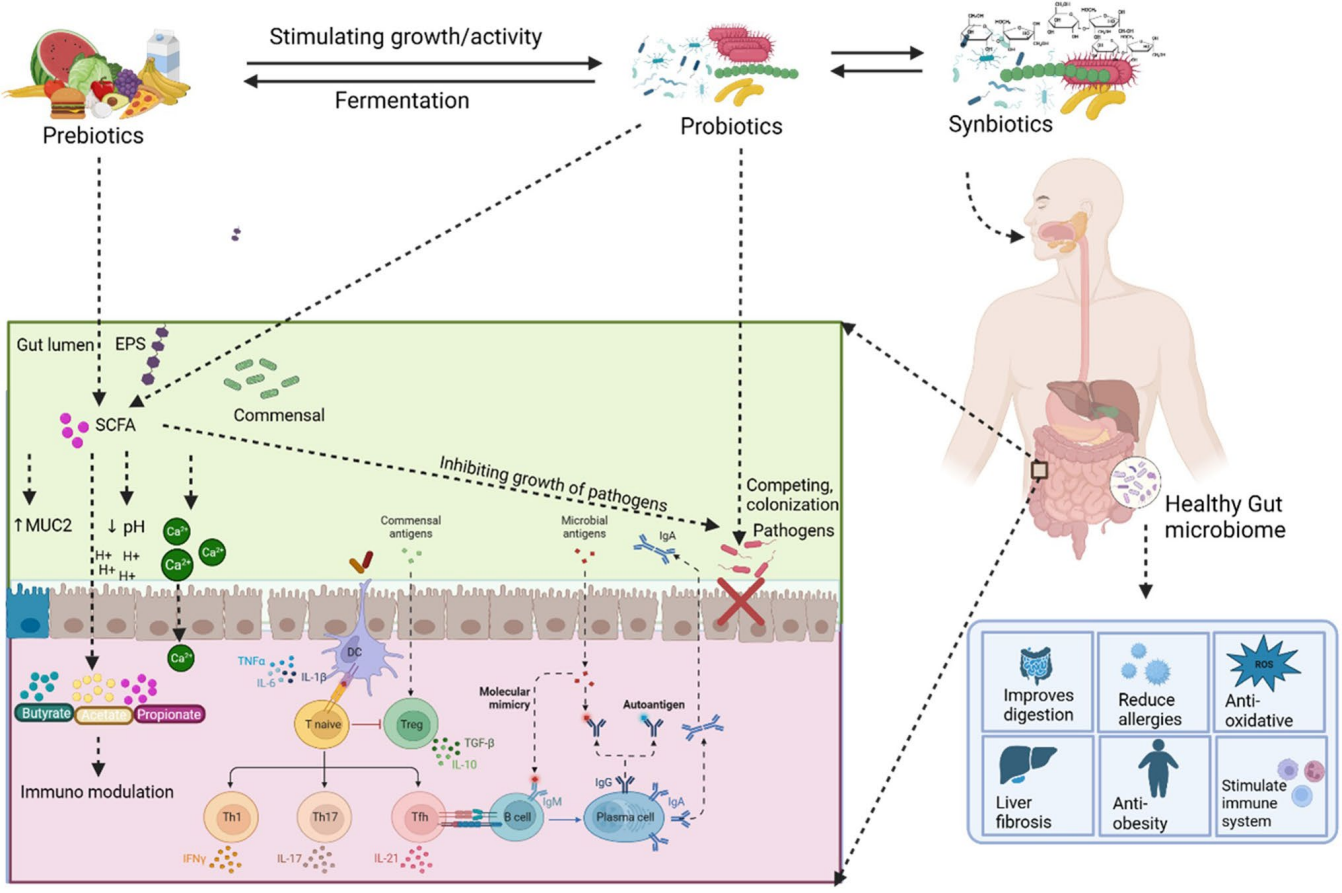
Schematic representation of prebiotics, probiotics, and synbiotics on healthy gut microbiome

In general, in the human GIT, prebiotics resist digestion in the small intestine due to a lack of enzymes. They travel intact in the large intestine and get degraded by gut flora resulting in fermented secondary metabolites. Promotion of beneficial microbes and fermentation of prebiotics contribute energy to the host by producing nutrition with the help of *Bifidobacterium* and *Lactobacillus.* Prebiotics affect intestinal microbiota and its metabolic activity by improving intestinal barrier function, resisting pathogens, increasing absorption of minerals, and lowering blood lipid levels as depicted in Fig. 4 [80]. Studies demonstrated that *Bifidobacterium* ferments the starch and other fructans [200],Van Den [247], breaking them into potential metabolites, including SCFAs (e.g., acetate, butyrate, and propionate), organic acids (e.g., lactate, succinate, and pyruvate), and gases, leading to a reduced pH and decrease in nitrogenous end products and fecal enzymes [122].

SCFAs have a pivotal impact on gut barrier integrity, mucus production, and regulating tight junctions. Numerous studies demonstrated their influence on glucose regulation, blood pressure, inflammation, and gut health. Moreover, SCFA also impacts energy balance, lipid metabolism, and immune function [173, 188]. In particular, SCFA has been shown to enhance the development and growth of goblet cells, which secrete mucins. SCFAs like butyrate have been shown to enhance mucin genes, increasing the expression of MUC2 glycoprotein. Also, it influences the glycosylation of mucins. A thicker mucus layer acts as a physical barrier against harmful substances, pathogens, and gut lumen particles [189, 265]. In immunomodulation, prebiotics can directly influence the host’s immune system and signaling pathways. Oligosaccharides, for example, increase the serum concentration of immunoglobulins (IgA, IgG, and IgM). They interact with carbohydrate receptors and activate specific immune responses. In cytokine modulation, inulin, XOS, and FOS influence the production of cytokines, including IFN-γ, IL-1β, and IL-10. GOS increases the IL-10 and reduces pro-inflammatory cytokines. SCFA regulates regulatory T cells (Treg cells), and gene expression related to cytokine production. Butyrate inhibits NF-kβ and activates PPARγ, resulting in the adhesion of immune cells to endothelial cells [274] [39, 201].

In cross-feeding, in the study Murakami et al. [169], alginate boosts the *Faecalibacterium prausnitzii*, facilitated by cross-feeding with specific Bacteroides strains. A study on healthy volunteers who were administered FOS and GOS found a reduction in “butyrate producers” levels and an increase in levels of *Bifidobacterium* [139]. A beneficial effect of oat-beta glucans in chronic gastritis in patients resulted in reduced mucosal damage. They determined lactic acid bacteria and SCFAs in feces [81]. In chelating and binding, some prebiotics can bind to certain minerals and make them soluble to absorb. For example, inulin and FOS can bind to minerals like calcium and magnesium, preventing them from forming insoluble compounds with other dietary components [43, 263].

#### Evidence for Effectiveness and Clinical Trials

Prebiotics, when used alone, cannot provide therapeutic effectiveness. They are known to increase the abundance of gut microflora that fights against many allergies and pathogens. In clinical examination of various prebiotics, for instance, clinical evaluation of inulin type fructan in the pediatric population reveals that it enhanced the population of Bifidobacterium and Lactobacillus in the gut [144]. Clinical trials investigated the effectiveness of prebiotics, demonstrating promising outcomes in gut health. For instance, clinical studies in Table 2 have revealed an increased population of beneficial bacteria like *Bifidobacteria* and *Lactobacilli*, while reducing the levels of harmful microbes. Several other oligo saccharides also showed a marked increase in the floral microbiome, thus coming in handy for treating various diseases. Clinical evidence also proved that prebiotics can contribute to digestive health by alleviating symptoms of conditions like IBS and constipation as summarized in Table 2.

### Synbiotic

A blend of live microorganisms and specific substrates that are preferentially utilized by beneficial host microorganisms results in advantageous effects on the host’s health. Limiting synbiotics to just a combination of probiotics and prebiotics might prevent the development of new synbiotics that are intended to work in concert [229]. Inducing prebiotic biosynthesis within the probiotic for synbiotic self-production or autologous synbiotics is a new method of their development [172]. As “synbiotic” indicates synergy, the term should only be used to describe products that a probiotic is preferentially favored by a prebiotic component microorganism. Therefore, a proper combination of both elements into a single product could assure a better outcome than either the probiotic’s or prebiotic’s activity alone. Synbiotic products with bacteria from the *Bifidobacterium* or *Lactobacillus* species and FOS appear to be the most commonly used [153]. Multidrug-resistant organisms are becoming increasingly widespread, which presents a special challenge to the world’s healthcare systems. Innovative strategies like synbiotics are required to stop the spread of infection caused by these organisms [171].

#### The Rationale Behind Synbiotics

Synbiotics, a combination of probiotics and prebiotics, significantly influence the gut microbiome and improve host health benefits [83]. These beneficial bacteria maintain the gut microbiome, digestive health, and mental health. On the other hand, dietary non-digestive carbohydrates and fibers nourish and stimulate the growth and development of these beneficial bacteria in the gut, promoting colonization and activity in the gut [85]. From the commercial and clinical perspective, synbiotics has more advantageous health benefits for consumers. Synergistic synbiotics can improve the development and persistence of beneficial microbes and may enhance health outcomes compared to individual probiotics and prebiotics [177]. They are effective in non-responders, thereby promoting clinical efficacy in consumers. Most clinical studies rely on microbiota analyses in gut microbiome research. Certainly, the production of effective prebiotics, probiotics, and synbiotics can deliver health benefits [76].

#### Benefits of Combining Prebiotics and Probiotics

An intriguing approach in the all-encompassing nutritional strategy to combat obesity is using synbiotics, and probiotic supplements incorporating prebiotic components. Supplementation with synbiotics raised the number of gut bacteria linked to good health, particularly *Bifidobacterium* and *Lactobacillus*, and it also seemed to improve the diversity of the gut microbiota. Dysbiosis of the gut microbiota has been shown to be a potential pathogenetic element in the emergence of polycystic ovary syndrome (PCOS). Prebiotic, probiotic, and synbiotic treatment in PCOS women has been demonstrated to enhance numerous biochemical outcomes and positive influence [280]. In treatments for inflammatory bowel syndrome (IBS) in children using synbiotics, it is advised to administer synbiotics twice daily [12]. In long-chain polyunsaturated fatty acids, and milk fat globule membranes, synbiotics administered in breastfed infants promoted the growth of probiotic and retarded gut microbiota maturation (Cerdó et al., 2022a). Synbiotics have beneficial effects on humans: maintaining intestinal microbiota balance [57]. The use of probiotics (*L. bulgarius* and *L. rhamnosus*) or prebiotics (XOS and RGE) accelerated recovery of antibiotic-damaged intestinal microbiota (C. [134, 135]). Improved immunomodulation by synbiotics alters the colonic gut microbiota, reduces gut-mucosa inflammation, and in treatment of immune-related diseases [202]. Some studies suggest that synbiotics can alleviate various GI diseases like IBS, constipation, diarrhea, and IBD [8, 40, 54, 249]. Synbiotics can also influence positively metabolic parameters such as lipid profiles, blood sugar levels, and body weight. This can be beneficial for patients with type 2 diabetes or metabolic syndromes [111, 113].

#### Evidence for Effectiveness and Clinical Trials

Synbiotics are preferable to using probiotics and prebiotics alone. Several previous findings have reported that the combination is beneficial in two ways: prebiotics help the growth of good microflora and eliminate the pathogenic ones. Some prebiotics also help release the SCFA, allowing the probiotics to grow and act longer. Some studies suggest that synbiotics can alleviate various GI diseases like IBS, constipation, diarrhea, and IBD [8, 40, 54, 249]. Synbiotics can also influence positively metabolic parameters such as lipid profiles, blood sugar levels, and body weight. This can be beneficial for patients with type 2 diabetes or metabolic syndromes [108, 151]. Most of the probiotics approved for clinical usage are the synergistic types with the prebiotics. Recently, In India, a synergistic combination of *Bifidobacterium breve* Bif11 combination with Iso-malto oligosaccharide (IMOS) has shown beneficial effects in treating ulcerative colitis (UC) [219]. Many synbiotics have been approved as therapeutic interventions in inflammatory diseases like UC, asthma, and IBD, which are listed in Table 2.

## Other Natural and Biological Products

### Herbal Remedies and Botanical Extracts

The earliest use of medicinal herbs, traditional Chinese medicine, green medicine, and botanical extracts as a part of food and pharmaceutical formulation dates back 5000 years ago being most advantageous worldwide [112, 272]. The practice of exercising the herbal constituents as part of therapy is termed Traditional Herbal Medicine (THM) [272].

#### Common Herbs and Botanicals Used for Gut Health

Many pharmaceutical formulations like ointments, pastes, and tinctures have been taken from the entire or specialized plant parts [272]. Few of the Food & Drugs Administration (FDA) approved phyto-organic compounds like digitoxin, vincristine, artemisinin, colchicine, and paclitaxel have been engaged as therapeutics in quite a lot of diseases [282]. Round about every aspect of our system by eclectic factors like diet, pathogens, stress, disease, and phytochemicals sway the structural community of the intestinal microbiome. These together illustrate an interplay between the intestinal microbiome and the phytochemicals [51, 282]. The interplay of herbs and microbiome health includes the augmentation of the bioactivity of the herbal medicine by biotransforming to their respective active metabolites. In addition to this, gut microbiome exhibits an explicit synergistic interaction with several chemicals and herbal medicines,likewise, herbal medicine promotes the shooting up gut microbiome composition [271].

**Fig. 5:**
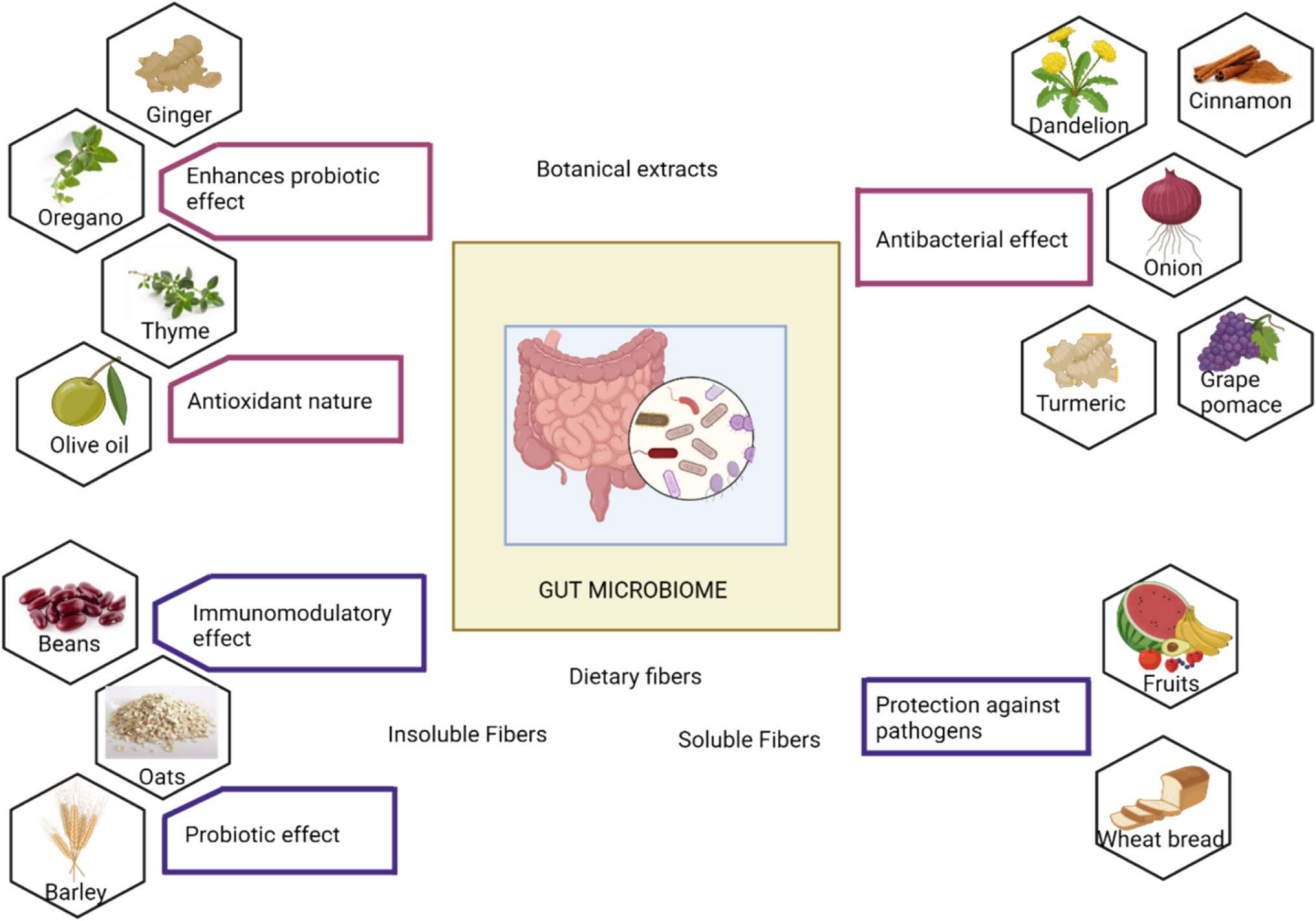
Schematic representation of botanical extracts and dietary fibers on gut microbiome

Several of the herbs and botanical extracts exercise their effects in safeguarding and strengthening the well-being of intestinal bacteria, as shown in Fig. 5. The thymol and carvacrol-containing oregano oil obtained from a culinary herb *Origanum vulgare* L. has shown evidence of antimicrobial, antioxidant, and anti-inflammatory expression when co-administered with probiotics. This combination improved gut health by hampering the growth of detrimental microbes in turbot (*Scophthalmus maximus*) in 30 days devouring experiment [79]. Thyme (*Thymus vulgaris* L.) is an aromatic polyphenol-constituted herb known to alleviate NLRP3 inflammasome-induced intestinal barrier damage. This action is associated with elevated *Faecalibaculum*, *Bacteroides*, *Romboutsia*, and *Blautia* populations coupled with the short-chain fatty acid (SCFA) synthesis, followed by downregulating the destructive bacteria [289]. Katharina et al. have demonstrated the significance of Wild Thyme (*Thymus serpyllum* L.) extract (WThE) on the gut microbiome in clinical subjects by assessing fecal microbial changes with elevated Firmicutes-to-Bacteroidetes ratio [121]. Studies focused on the phenolic compounds obtained from olive oil when administrated with thyme polyphenol extract have been demonstrated to elevate the Bifidobacterial population and decline the oxidized LDL-C levels in cases of hypercholesteremia [156]. Rosemary (*Rosmarinus officinalis* L.) centric studies having 40% carnosic acid (CA) illustrated a synergistic antiobesity activity in association with the microbial community by decreasing Firmicutes to Bacteroidetes ratio. It was further noticed an increasing primacy of functional and probiotics bacteria, including *Clostridium innocuum, Muribaculaceae unclassified*, and *Akkermansia muciniphila*, and inhibited *Firmicutes* and *Proteobacteria* in C57B/L6 mice fed with high-fat diet holding 0.1/0.2% carnosic acid [87]. Studies focusing on essential oils validated that rosemary oil displays a prebiotic effect via increasing the *Lactobacillus* genus of the microbial community [208]. Colorectal cancer is often associated with gut dysbiosis. Studies have demonstrated that upon treatment with different concentrations of carnosic acid, elevated levels of anti-inflammatory bacteria like Bifidobacterium, Blautia, Faecalibacterium, and Subdoligranulum were demonstrated, along with decreased levels of Bacteroides, Desulfovibrio, and Bacteroides alluding the prominence of gut microbiome in anticancer effect (S. [137, 138]). DSS-induced colitis mice fed with cinnamaldehyde-enriched cinnamon essential oil (CEO) enhanced the intestinal microbial community via increasing the Alloprevotella and Lachnospiraceae _NK4A136_ group that fabricate the SCFA. Additionally, a marked decrease in Bacteriodes and Heliobactor known for their negatively and positively associated TLR4 and tumor necrosis factor-α activity was recorded (A. li [134, 135]). Polyphenol-centered studies marked for reduced fat mass gain, steatosis, and glucose homeostasis reasoned by the altered gut microbiome activity were validated with the administration of cinnamon bark extract (CBE) with a high-fat diet in C57B/L6 mice. This study reported promising results with decreased Peptococcus genus by CBE and grape pomace extract (GPE), specifically improving Roseburia and Allobaculum, along with decreased Lactococcus and Disulfovibrio species [250]. Another study focused on cinnamon uptake which enhanced altered gut microbiota portrayed the decreased bacterial class called Gammaproteobacteria in the large intestine (J. I. [118, 119]). The Grape Seed constituted Proanthocyanidin extract (GSPE) improves gut health by collectively increasing Verrucomicrobia and Akkermansia and Firmicutes-to-Bacteroidetes ratio in dextran sulfate sodium (DSS) induced colitis C57B/L6 mice via downregulating the proinflammatory cytokines action [220]. Licorice (*Glycyrrhiza glabra*) reshaped the gut microenvironment in studies where all doses of licorice extract shrunk the Lachnospiraceae_NK4A136_group and high-dosed licorice improved the Akkermansia, Bacteroides, and Alloprevotella [283]. Licorice ethanol extract (LEE) unveiled improved intestinal dysbiosis by shrinking the Firmicutes/Bacteriodetes ratio and increasing anti-obesity-associated bacteria in a study where C57B/L6 male mice were fed with high fat diet (HFD) (F. [140, 141, 143]). A garden weed named Dandelion scientifically termed *Taraxacum officinale* was known for its pharmacological GI-protective effects by way of normalizing the gut microbial microenvironment. This weed with its triterpenes, sesquiterpenes, and complex carbohydrates dosed at 0.5–1 g/kg bodyweight hepatic steatosis modeled rat decreased the Firmicutes/Bacteriodetes ratio alleviating gut dysbiosis (Y. [137, 138]). Dandelion extract demonstrated improved gut microbiome diversity, increased abundance of Firmicutes and Actinobacteria, and reduced detrimental bacteria in Golden Pompano at a dietary dose of 0.1–1 g/kg [231]. The dietary Dandelion (*Taraxacum mongolicum*) polysaccharides (DP) were portrayed to transform the caecum microbiota of a laying hen by increasing the SCFA and boosting the relative abundance of Romboutsia, Alloprevotella, and Parabacteroides in the cecum [30]. A study focused on a rhizome named Ginger (*Zingiber officinale*) perineal natural product disclosed a marked increase in gut microbial species of the genus Bifidobacterium and SCFA-producing Alloprevotella and Allobaculum bacteria that displayed anti-obesity therapeutic effects in HFD fed mice (J. [257, 260]). Ginger extract demonstrated beneficial effects in antibiotic-associated diarrhea (AAD) by decreasing the Escherichia_Shigella increasing the Bacteroides population and regulating the intestinal barrier integrity [149]. There were studies supporting the regulation of the gut microenvironment in DSS-induced ulcerative colitis by ginger polysaccharides (GP) [86]. GP (UGP1, UGP2) modulated gut microbiome by reducing Firmicutes/Bacteriodetes ratio, Bacteriodetes, Verrucomicrobia at the phylum level, Bacteriodaceae, Muribaculaceae, and Lactobacillaceae at the family level with decreased Lachnospiraceae and Rikenellaceae families thereby enhancing intestinal immunity (J. ping [140, 141, 143]). Ginger-focused studies demonstrated a dwindle in Ruminiclostridium_sp_KB18 and Lachnospiraceae _bacterium_615 and a surge in Muribaculaceae in DSS-induced colitis mice [82]. Ginger essential oil and citral therapy improve the atherosclerotic condition in ApoE^−/−^ mice by squelching cardiovascular disease-associated gut microbiota like Enterorhabdus and Proteus. An increase in the amount of Allobaculum and Akkermansia ceasing the inflammation and endotoxemia-induced atherosclerosis was noticed [178].

#### Mechanisms of Action and Potential Benefits

The earlier statements strongly validate that natural and biological products have emerged as promising interventions to prevent gut dysbiosis and stimulate the gut microenvironment. Understanding how natural and biological sources affect the gut microbiome is essential for effectively harnessing their potential. This section will dwell on various mechanisms of action exerted by these natural and biological products.

a) Prebiotic Effect

Natural and biological products exhibit substantial prebiotic effects on the intestinal microenvironment suggesting their prominence in regulating gut dysbiosis. Studies conducted on garlic (*Allium sativum*) signified that fructans ideally function as the prebiotic effects [35]. In vitro gut microbial modulation studies on cooked legumes like Black bean (*Phaseolus vulgaris*) and Cowpea seeds (*Vigna unguiculata*) containing excessive amounts of bioactive polyphenolic constituents demonstrated prebiotic potential by decreasing gut pH significantly and increasing SCFA metabolism [236].

b) Antibacterial Effect

The evidence of the antibacterial effect of natural and biological products on gut microbiome was procured from several studies. Natural sources like garlic (*Allium sativum*) exhibit an antibacterial effect from their oil-soluble organosulfur constituents like allyl sulfides, allicin, and ajoenes; particularly, allicin disrupts bacterial cell membranes [17]. Ajoenes promote biofilm killing, and terminate lysis of polymorphonuclear leukocytes, and anti-quorum sensing action [103]. Ginger (*Zingiber officinale*) with shogaols and gingerols exhibits a strong antibacterial action by preventing the multiplication of digestive microbes [273]. Li et al.’s studies on curcumin demonstrated inhibitory action on metabolism and biofilm formation, carbohydrate metabolism, and extracellular lipopolysaccharide synthesis of *Streptococcus mutans* the causative species for dental caries after both a 5-min and a 24-h time period [102]. Dietary antibacterial-rich polyphenolic extracts exhibit distinct antibacterial effects in gram-positive and gram-negative bacteria by altering the membrane permeability along with enhancing the bacterial sensitivity to antibiotic therapy [222].

c) Antioxidant Nature

Green tea polyphenols like catechins exhibit free radical scavenging activity along with an antioxidant effect [181]. *Cinnamomum cassia* leaf filtrates demonstrated an antioxidant effect by increasing glutathione peroxidase and superoxide dismutase along with inhibiting the formation of malondialdehyde in the gut microenvironment [36].

#### Evidence of Effectiveness and Clinical Studies

Clinical studies focused on the modulatory effects of herbal and natural products on gut microbiome signify their effectiveness in improving microbiome activity. A randomized double-blind clinical trial of curcumin extract on GI symptoms and gut microbiome depicted an increase in the intestinal microbial species with a 69% increase with curcumin and 7% with turmeric [146]. In a randomized study, T2DM patients with hyperlipidemia were given either metformin or Chinese herbal formula where both formulations improved glycemic and lipid profiles by increasing the *Blautia* spp. Apart from that the Chinese herbal formula also improved the *Faecalibacterium* spp. enrichment associated with an altered triglyceride profile [242]. Nutritional care (NC) gut relief formula containing guar gum, glutamine, Aloe vera, pectin, peppermint oil, curcumin, and slippery elm revealed the significance of botanical extracts in the downregulation of the GI symptoms like constipation, indigestion, diarrhea, nausea, abdominal pain, heartburn, and troublesome flatulence, by stabilizing specific gut microbiome [197]. The Huayu-Qiangshen-Tongbi (HQT) TCM when administrated to patients with rheumatoid arthritis (RA) modulated the abundance of gut microbes as enlisted in Table 3 [158]*.* Aronia polyphenol extract was proven to improve arterial function in prehypertensives by increasing the abundance of butyrate-producing gut bacteria [130]. Cranberry extract is a rich source of polyphenols and was hypothesized to minimize the gut microbe homeostasis featured in chronic kidney disease (CKD) [235].

**Table 3:** Summary of clinical study reports of herbal/botanical extracts on the gut microbiome

Herbs/botanical extracts	Study duration and dose	Number of subjects	Clinical study design	Microbiome modulation	Ref
Curcumin	8 weeks, 500 mg	79 adults	Randomized, double-blind, placebo-controlled study	Not specified	[146]
Metformin and traditional Chinese medicine	12 weeks, 250 g/time or 3times/day and 1unit/day	450 T2DM patients	Multicenter, randomized, open-label	Increase *Blautia* spp., *Faecalibacterium* spp.	[242]
NC gut relief formula	16 weeks, 5 g/day followed by 10 g/day for 4 weeks	43 participants	Single arm pre-post case study	Increase *Lactobacillus, Clostridium*, and *Faecalibacterium prausnitzii*	[197]
HQT decoction	6 months, once every 2 days	22 Controls, 22 RA patients	Case–control study	Increase *C. somerae*, *Haemophilus* spp., *Dialister* spp*.*, *C.celatum, Roseburiaw* spp.*, Turicibacter* spp*., Pasteurella bettya* Decrease *Clostridium symbiosum,* and *Clostridiales bacterium 1–7-47FAA*	[158]
Aronia berry extract	12 weeks	51 controls and 51 prehyper-tensive patients	Randomized double-blind placebo-controlled study	Increase *Lawsonibacter asaccharolyticus* and *Intestinimonas butyriciproducens*	[130]
Cranberry extract	2 months 500 mg 2times/day	25 CKD patients	Randomized double-blind placebo-controlled study	Not specified	[235]

### Dietary Fibers and Resistant Starches

Eben Hipsley in the year 1953 coined the term “Dietary fibers” to define the indigestible plant cell wall constituents. This was redefined by Trowell and team as “plant cell remnants residing within ileum, resistant to gut enzymes, but partially hydrolyzed by colonic bacteria” [50]. In the recent scenario, three major organizations which include the American Association of Cereal Chemists, the Codex Alimentarius Commission, and the Institute of Medicine (now Health and Medicine Division) of the National Academy of Sciences, Engineering and Medicine defined dietary fibers. The American Association of Cereal Chemists defines dietary fibers as edible plant products that are not hydrolyzed by the small intestine but partially/completely fermented by the large intestine. The Codex Alimentarius Commission defines dietary fibers as edible carbohydrate polymers of 10 or more monomeric subunits that are non-digested by intestinal enzymes. Finally, the Institute of Medicine delineated the non-digestible combination of lignin and complex carbohydrates as dietary fibers [124]. Non-digestible dietary fibers are believed to be a major substrate for the gut microbiome of which fructo-oligosaccharides and inulin explicitly benefit the host microbiome [187].

#### Importance of Fiber in Gut Health

The diversified gut microenvironment, composition, and richness greatly influence physiology. Dietary fibers are considered the essential components in a balanced diet enhancing overall health by regulating the gut microbiome composition [148]. Fiber fermentation products in the colon were postulated to promote suppressing carcinogenesis and colonic inflammation [175]. Studies focused on the role of dietary fibers stated the modulatory role of these on the murine gut microbiota via regulating the microbiome diversity by promoting the production of SCFA [233]. Certain dietary fibers serve as prebiotics also called microbiota-accessible carbohydrates (MACs) via upholding specific probiotic proliferation [77].

#### Types of Dietary Fibers and Resistant Starches

Dietary reference intake (DRI) deliberations classified fibers into three groups: (i) dietary fibers (oat bran, wheat), (ii) functional fiber (resistant starches), (iii) total fiber [116]. According to Codex Alimentarius Alinorm in 2009, these fibers are classified based on their source as (i) carbohydrate polymer from cereals, vegetables, and tubers,(ii) synthetic carbohydrate polymer like methylcellulose; and (iii) edible carbohydrate polymer obtained from raw foods by enzymatic and physiochemical means. Apart from the source, based on the structure of carbohydrate polymer, the dietary fibers were also classified into three subgroups termed (i) resistant oligosaccharides, (ii) resistant saccharides, and (iii) non-starch polysaccharides centered on their structure [78, 107, 116, 152]. Dietary fibers are also reclassified based on their solubility as soluble dietary fiber (SDF) that includes water-extractable arabinoxylans (AX), GOS and FOS, β-glucans, arabinoxylan oligosaccharides (AXOS), and insoluble dietary fiber (IDF) like resistant starch (RS), lignin, cellulose, and water-unextractable AX [148].

RS is one of the types of dietary fibers that are grouped on the basis of their resistance to host enzymes as resistant starch type 1 (RS1)—physically impenetrable trapped within partially or completely grounded grains; resistant starch type 2 (RS2)—raw starch grains (potato and banana); resistant starch type 3 (RS3)—retrograde starch; resistant starch type 4 (RS4)—chemically modified enzyme resistant starch (starch esters, starch ethers, cross-linked starches); and resistant starch type 5 (RS5)—starch lipid-rich complexes [64, 84, 261].

#### Mechanisms of Action and Benefits in Promoting a Healthy Gut Microbiome

The dietary fibers are indigestible plant-based carbohydrates associated with the protection and maintenance of gut microbiome along with many health benefits.

a) Prebiotic Effect

As stated in the earlier section, prebiotics promote the selective growth of the gut microbiome. The characteristic prebiotic nature of fermentable fibers like hemicellulose, oligosaccharides, gums, β-glucan, and RS function as substrates for microbes producing SCFA-like butyrate, acetate, and propionate [29, 96]. Dietary fiber provides nutritional niches to gut microbes where the administration of resistant starches encourages the growth of *Eubacterium rectale*, *Bifidobacterium adolescentis*, and *R. bromii* [50]. A high-fiber diet increases the diversity of the gut microbiome which improves the metabolic and immune function thereby reducing the risk of gastrointestinal diseases [225]. Prebiotic dietary fiber like inulin promotes the growth of beneficial microbes like *Bifidobacterium*, *Lactobacilli*, and *Streptococci* species at the expense of pathogenic ones [234]. RS upon fermentation also reduces the colonic pH by SCFA production and downregulates the secondary bile acid secretion, ammonia, and phenol concentration within the colon. These RS act as capsulation substances for probiotics enhancing their survival against the harsh gastrointestinal pH [22].

b) Resistance Against Pathogenic Microbes

Dietary fibers enhance resistance against pathogenic organisms by promoting the production of SCFA from fiber fermentation, decreasing intestinal pH, and impairing the overgrowth of pH-sensitive pathogenic species [253]. In vitro and In vivo studies focused on gut microbiome justified the acidification of the colonic environment by SCFA-like propionate kills pathogenic microbes like *Salmonella* and *E. coli* [243]*.* SFCA maintains the dynamics of the intestinal epithelium along with influencing electrolyte reabsorption, gut motility, and immune activity epigenetically [187].

c) Immunomodulatory Effect

The probiotics consume the prebiotic dietary fibers as their food source that are further fermented to produce SCFA secrete mucus. The prebiotics interaction with probiotic membrane-bound toll-like receptors stimulates the immune response [190]. The dietary fibers stimulate the activation of Treg cells by epigenetic modulation and inhibit the inflammatory dendritic cells by transcriptional regulation. Non-starch dietary fiber like pectin fermented by probiotics like *Bacteroides* and *Firmicutes*. Pectin upon esterification inhibited induced nitric oxide synthetase (iNOS) and cyclo-oxygenase 2(COX2) in LPS-activated murine macrophages along with inhibition of NFкB, MAPK, and AP-1 pathways [34].

#### Evidence of Effectiveness and Clinical Studies

The aforementioned statements give the significant role of dietary fibers in the gut microbiome which was further justified in clinical studies. Studies focusing on the bacterial 16S rRNA sequencing analysis have demonstrated the presence of SFCA-producing bacteria like *Bacteroidetes*, *Actinobacteria*, *Xylanibacter*, and *Prevotella* in rural African children taking legume fiber and an agrarian diet rich in fruit. They also postulated the decrease in the relative abundance of pathobionts like *Escherichia* and *Shigella* [49]*.* Nielson and the team demonstrated the dietary intervention of resistant starches on gut microbiome and SCFA dynamics. They assessed that resistant starches from maize (RMS), potatoes (RPS), and inulin from chicory root greatly increase the SCFA, particularly butyrate production, in addition to this increase in *Bifidobacteria*, *Clostridium chartatabidum*, and *Ruminococcus bromii* species [15]. In 2018, Liping et al. outlined the role of dietary fibers in alleviating type 2 diabetes mellitus (T2DM) by selective promotion of the gut microbiome. They randomized the patient population as the control group (patients receiving normal care) and treatment group (received prebiotics, high fiber diet, and Chinese traditional medicine) where they postulated that the treatment group exhibited the increased growth of butyrate and acetate-producing bacteria with a reduction in HbA1c values [285]. Studies focused on the immunomodulatory effect of the intestinal microbiome with malignancies like melanoma stated that the improved progression-free state in 128 patients on immune checkpoint blockade (ICB) had dietary fiber supplementation by modulating the gut microbiota [227]. As stated in Table 4, Physillum husk when administered to constipated patients posed a greater modulation of the gut microbiome resulting in altered SFCA levels [104]. The prominence of dietary fibers in the metabolic regulation of gut microbiome was specified in a clinical study by dietary intervention with white wheat bread (WWB), and barley kernel bread (BKB), which correlated the *Prevotella* to *Bacteroides* ratio to glucose metabolism [125, 210]. The prebiotic effect of whole-grain (WG) and wheat bran (WB) cereals was illustrated in a clinical study that reported an increase in *Bifidobacteria*, and *Lactobacilli* species in WG group compared to WB group [44]*.*

**Table 4:** Summary of clinical study reports of dietary fibers and resistant starches on the gut microbiome

Dietary Fibers/Resistant Starches	Study duration and dose	Number of subjects	Clinical study design	Microbiome modulation	Ref
Dietary fiber	African children 10 g/day (1–2 years) 14.2 g/day (2–6 years) European children 5.6 g/day (1–2 yr) 8.4 g/day (2–6yrs)	29 children	Randomized trial	Increase *Bacteroidetes, Actinobacteria*, *Xylanibacter*, and *Prevotella* Decrease *Escherichia* and* Shigella*	[49]
Resistant starches	2 weeks, 28–34 g/day RPS, 20–24 g/day RMS, inulin 20 g/day	147 healthy subjects	Randomized interventional study	Increase *Bifidobacteria*, *Clostridium chartatabidum*, and *Ruminococcus bromii*	[15]
High fiber diet	Not specified	43T2DM patients	Open-label parallel randomized study	Increase *Bifidobacterium, Faecalibacterium prausnitzii,*	[285]
Dietary fiber	5 g/day	438 melanoma patients	Large cohort study	Increase Ruminococcaceae family and *Faecalibacterium* genus	[227]
Physillum husk	7 days	8 controls, 16 constipat-ed patients	Randomized case–control study	Increase *Lachnospira*, *Faecalibacterium*, *Phascolarctobacterium*, *Veillonella*, and *Sutterella* Decrease *Coriobacteria* and *Christensenella*	[104]
WWB, BKB	3 days, WWB10.7 g/day, BKB 36.4 g/day	99 healthy subjects	Randomized study	*Prevotella/Bacteroides* ratio not prognostic of metabolic response	[210]
BKB	3 days, BKB 37.6 g/day	39 healthy subjects	Randomized cross-over study	Increase *Bacteroidetes* and *Prevotella/Bacteroides ratio*	[125]
WB, WG	2 × 3 weeks with 2-week intervals between, WB 48 g/day, WG 48 g/day	31 healthy subjects	Placebo-controlled, double-blind cross-over study	Increase* Bifidobacteria, Lactobacilli*	[44]

## Safety and Considerations

### Safety Profile of Natural and Biological Products

Safely using natural and biological products is a primary requisite to ascertaining these products’ safety profile. There is no defined assurance for the safety profile of these natural and biological products. As stated in the earlier sections, natural and biological products positively affect gut health. The safety profiling of specified microbial species can be defined based on bacterial pathogenicity, identity, a complete set of knowledge, and application [230]. As stated in the earlier sections, studies focused on probiotic intake have shown a positive safety profile. The safety profile for probiotics includes the consideration of patient liability, duration, dose, frequency, and route of administration [211]. However, there is evidence of the effectiveness of probiotics on gut health, but few studies have demonstrated transmissible antibiotic resistance and enterotoxin production with the species of enterococci and *Bacillus cereus*, respectively [230].

#### Regulation on Prebiotics and Probiotics Safety and Novelty

While “safety” encompasses scientific and regulatory aspects, “novelty” pertains to regulations. Novel probiotics and prebiotics are treated the same as non-novel substances. Safety evaluation is complicated and requires experiential knowledge and planned research. On one hand, food substances have been part of the human diet for ages without harm. Some foods have centuries of safe use with minimal scientific review. The examples are from Title 21, Part 182 of the US Code of Federal Regulations, which lists “Substances Generally Recognised as Safe.” Advanced substances are assessed for safety using historical common use and rigorous scientific scrutiny. QPS and US GRAS microorganisms are examples. Novel substances require extensive scientific evidence for safety assessment, but lack historical usage [129]. The global regulations for novel, functional, and traditional foods vary worldwide. Novel foods not used in the EU before 15 May 1997 are regulated by the 1997 Novel Food Regulation 285/97/EC. It is apparent in this law that innovative foods must undergo risk assessment before being in the market in EU. The legislation allows a simple notification process for substantial equivalence to regularly used foods. A proposed new version was published in December 2013 by revising the 1997 Novel food regulation [129, 214]. In Canada, “Novel food” and “major change” are defined in the Food and Drug regulations section B.28.001. Microbe-derived products need safe use history, new process details, genetic knowledge, and food use. The safety of new eligible probiotics is comparable to European QPS bacteria. Food and Drug regulations classify therapeutic claims as “drugs” and regulated as per the Food and drug regulations documented in Health claims-Probiotic claims–Agency CFI [129]. In the USA, all food and food ingredients are regulated under the Food, Drugs and Cosmetic Act (FDCA). In the USA, Food manufacturers are responsible for new food safety. The regulation requires FDA premarket assessment and clearance for any substance intentionally added to food unless experts consider it safe under its intended usage conditions. Prebiotics and Probiotics in non-supplement foods are regulated like other food ingredients in the USA. They can be described as food additives or GRAS comp at the manufacturer’s discretion (Arturo [7]. With the increasing number of probiotics and associated health claims, thorough safety assessments are crucial before human consumption. Kumar et al. recommended scientific information required for novel probiotics in addition to the mentioned WHO/FAO guidelines 2002. The following recommendations are as follows: (a full genome sequencing and annotation predict function,(b antibiotic resistance profiles and resistance types should be characterized, including conjugation studies; (c toxicological testing to guarantee innovative probiotics are safe; (d defining target populations since probiotics may differentially affect subgroups; (e selection of in vivo models, recognizing mouse/rat models do not replicate human GI conditions but can provide preliminary testing for newly known strains (Henk [90]). These supplement WHO/FAO novel probiotic strain safety guidelines. For novel strains, scientific reviews are often needed to support health claims before sale [129].

### Potential Side Effects and Contraindications

The potential side effects of probiotics are majorly seen in diseased populations with the transfer of bacterial genes directed towards systemic infection and antimicrobial-resistant gene transfer to highly infective bacteria. Apart from the above-mentioned side effects, there is short- and long-term immune stimulation in immunocompromised populations, hospitalized patients, and unintended metabolic activities like lactic acidosis, and bile acid deconjugation [184, 230],Van Den [248].

#### Systemic Infections

Even though probiotics are effective in many diseases, there is evidence of commensal interactions resulting in fungemia and bacteremia [52]. Studies focusing on probiotic adverse effects in clinical subjects discussed the incidence of Fungemia who have consumed probiotic *S.boulardii* presenting bacterial strains of *Saccharomyces cerevisiae or Saccharomyces boulardii* in their blood samples due to digestive translocation [52, 56]. Patients administered with probiotics reported bacteremia associated with *Lactobacillus* species predominantly by *Lactobacillus rhamnosis* followed by *L. acidophilus* and *L. casei* [56]. Probiotic adherence to the intestinal mucosa fostering their translocation into the bloodstream can be the possible reason for bacteremia. There was clinical evidence in cases with diarrhea and vaginal candidiasis reporting the incidence of endocarditis in patients treated with probiotics like *Lactobacillus acidophilus* and *Lactobacillus paracasei* [114]*.*

#### Detrimental Metabolic Activities

Probiotics can vehiculate deleterious metabolic activities when colonized in large numbers within the small intestinal mucosa. They promote excessive bile acid deconjugation resulting in diarrhea and intestinal lesions. Strains of *Lactobacillus* and *Bifidobacterium* were reported to induce intestinal mucosal damage [155]. The PROPATRIA trial has speculated the incidence of intestinal ischemia in the pancreatitis subjects nursed with probiotics likely induced an inflammatory response. Another deleterious metabolic concern is the increased secretion of d-lactate resulting in lactic acidosis [56].

#### Immune System Activation

It was assumed that probiotics trigger both innate and adaptive immune systems on systemic administration by the release of peptide-glycan-polysaccharide, activating dendritic cells and secreting cytokines [56, 155].

#### Antibiotic Resistance

Another potential side effect of probiotics is the emergence of antibiotic resistance via the horizontal gene transfer of the resistance genes by probiotic *Bifidobacterium* and *Lactobacillus* to pathogens within the intestinal mucosa [160]. Studies focused on the *Lactobacillus* postulated resistance against macrolides, tetracycline, lincosamide, erythromycin, streptogramin, and chloramphenicol.

### Interactions with Medications and Other Supplements

Prebiotic (fructo-oligosaccharides) and probiotic-focused studies postulated their interactions with other medications. *Lactobacillus casei* probiotics increased the transport of drugs like β-lactam antibiotics, and angiotensin-converting enzyme inhibitors (ACE inhibitors) by escalating the activity of the oligopeptide transporter hPEPT1 in the small intestine [23]. The gut microbiome produces a significant amount of vitamin K and probiotics that alter vitamin K affecting anticoagulant sensitivity [254]. In the context of incubation of oral anticoagulants like acenocoumarol with probiotics like *Bifidobacterium*, it exhibited metabolic alternation due to bacterial enzymatic activity [207]. Adjuvant therapy of antipsychotics like selective serotonin reuptake inhibitors (SSRI) with probiotics demonstrated synergistic response in patients with generalized anxiety disorder (GAD) and major depressive disorder by increasing their tolerability thereby improving schizophrenic symptoms [63]. In the context of autoimmune diseases like rheumatoid arthritis, the combined therapy of probiotics (*Enterococcus faecium*) with methotrexate decreases the necessity for additional therapeutic measures compared to methotrexate alone [28]. Concurrent administration of antibiotics with probiotics may result in massive destruction of these microbes resulting in decreased efficiency of *Lactobacillus* and *Bifidobacterium* species. *S. boulardii* interact with antifungal reducing their efficiency [254].

### Recommendations for Use and Dosage Guidelines

The selection criteria for probiotics are quite essential, especially in high-risk populations [131]. The expert panel does not provide substantial recommendations for the use of probiotics for the healthy population. The metanalysis and published reviews along with studies on the use of probiotics for several diseases may provide a few insights into the dosage recommendations (“Probiotics—Health Professional Fact Sheet,” n.d.). World Gastroenterology Organization (WGO) recommends the use of probiotics in accordance with the strain and product. WGO recommends that clinicians should detail the use of the strain, the ideal dose for specific clinical indication, and the duration of use along with manufacturing guidelines (“WGO Probiotics and Prebiotics Guideline Summary,” n.d.). ISAPP provides the manufacturer guidelines for both probiotics and prebiotics to enlist the use by and expiry date on the label (“Probiotics—Health Professional Fact Sheet,” n.d.). The efficacious dose and the time of administration of specific strain of microbe is quite essential. A minimum dose of 10^6^–10^9^ colony-forming units (CFUs) per day is considered to be safe for human consumption [131]. The use of probiotics in the pediatric population for specified clinical conditions was characterized by a metanalysis report for clinical trial data. A dose of 10^8^–10^9^ CFU/day of *Lactobacillus rhamnosus* GG (LGG) decreased the occurrence of respiratory tract infections. For nosocomial diarrhea and urinary tract infection, LGG at a dose of 10^9^ CFU/day was advised to reduce the risk for either condition. In the case of acute gastroenteritis (AGE), LGG and *S. boulardii* were administrated together for 5–7 days at a dose range of ≥ 10^10^ CFU/day and between 250–750 mg/day respectively as an add-on to oral rehydration therapy. The probiotic *L. reutie* DM 17938 at a dose of 10^8^ CFU/day for 21–30 days was recommended as an effective therapeutic option in the prevention of infantile colic [95].

## Data Availability

No datasets were generated or analysed during the current study.

## Abbreviations

AXOs: Arabinoxylo-oligosaccharides
AMPs: Antimicrobial proteins
AAD: Antibiotic-associated diarrhea
AXOS: Arabinoxylan oligosaccharides
BKB: Barley kernel bread
CPs: Capsular polysaccharides
CA: Carnosic acid
CEO: Cinnamon essential oil
CBE: Cinnamon bark extract
CKD: Chronic kidney disease
COX2: Cyclo-oxygenase2
DP: Dandelion polysaccharides
DDS: Dextran sulfate sodium
FAO/WHO: Food and Agriculture Organization of United Nations and World Health Organization
FOS: Fructo-oligosaccharides
FOXs: Feruloylated arabinoxylo-oligosaccharides
FDA: Food and Drugs Administration
GI: Gastrointestinal tract
GF: Germ-free mice
GABA: Gamma-aminobutyric acid
GOS: Galactic-oligosaccharides
GPE: Grape pomace extract
GSPE: Grape seed constituted proanthocyanidin extract
GP: Ginger polysaccharides
HMOs: Human-milk oligosaccharides
HFD: High fat diet
HQT: Huayu-Qiangshen-Tongbi
IgA: Immunoglobulin A
ISAPP: International Scientific Association of Probiotics and Prebiotics
IFN-γ: Interferon-gamma
ITF: Inulin-type fructan
IBS: Inflammatory bowel syndrome
IMOS: Iso-malto oligosaccharide
IDF: Insoluble dietary fiber
iNOS: Induced nitric oxide synthetase
LAB: Lactic acid bacteria
LC-PUFA: Long-chain polyunsaturated fatty acids
LEE: Licorice ethanol extract
mLNs: Mesenteric lymph nodes
MAMPs: Microbial-associated molecular patterns
MFGM: Milk fat globule membranes
MACs: Microbiota-accessible carbohydrates
NEC: Necrotising enterocolitis
NC: Nutritional care
PRRs: Pattern recognition receptors
PCOS: Polycystic ovary syndrome
RA: Rheumatoid arthritis
RS1: Resistant starch type 1
SLPs: Surface layer proteins
SCFA: Short-chain fatty acids
SDF: Soluble dietary fiber
Tregs: T regulatory cells
Th17: T helper 17
TLRs: Toll-like receptors
TGF-β: Transforming growth factor-beta
TO: Trans-galacto-oligosaccharides
THM: Traditional herbal medicine
T2DM: Type 2 diabetes mellitus
UC: Ulcerative colitis
WThE: Wild thyme extract
AX: Water-extractable arabinoxylans
WWB: White wheat bread
WG: Whole-grain
WB: Wheat bran
XO: Xylooligosaccharides
